# Evaluation of the mechanical properties of porcine kidney

**DOI:** 10.1371/journal.pone.0307778

**Published:** 2024-07-25

**Authors:** Zhao Zhang, Xianglong Tan, Mengyang Li, Wubuliaisan M., Shangjian Zeng, Yanqing Wu

**Affiliations:** 1 State Key Laboratory of Science and Technology of Explosion, Beijing Institue of Technology, Beijing, China; 2 Department of Hepatobiliary and Pancreatic Surgery, The First Medical Center of the PLA General Hospital, Beijing, China; University of Saskatchewan, CANADA

## Abstract

With the development of medical diagnosis and treatment, knowing the mechanical properties of living tissues becomes critical. The aim of this study was to investigation material properties of the fresh porcine kidney and the parametric characterization of its viscoelastic material behavior. The material investigation included uniaxial tension tests in different strain rates, relaxation tests, as well as hydrostatic compression tests on the samples extracted from the fresh porcine kidney cortex. Tension tests and relaxation tests were performed by a planar dog-bone specimen with a micron loading testing machine. Hydrostatic compression tests were performed on the kidney cylinder sample which was placed in a compression chamber. Furthermore, a nonlinear viscoelastic model recently proposed by us was employed to characterize the tension data at different strain rates and relaxation test data. The the experimental and numerical results show that the stress-strain relations of the porcine kidney cortex at different strain rates in tension are presented for the first time and a higher strain rate results in higher ultimate strength and initial Young modulus but a lower rupture strain. A damage-dependent visco-elastic model is employed to model the tension data at different strain rates and relaxation data and exhibits a good agreement with the experimental data, which also demonstrates that the damage has an obvious influence on the stress-strain relation. Through comparison with the existing reference covering the uniaxial compression data, it seems that the mechanical behavior of the porcine kidney cortex manifests a stress state-dependent mechanical behavior. The ultimate strength and rupture strain are larger in compression than that in tension.

## 1. Introduction

Mechanical properties of living organs or issues have always been an important topic in the Biomechanics community. The mechanical properties of soft biological tissues are critical for many real applications, such as medical disease diagnosis [1, 2], virtual reality simulation [3, 4], trauma medical treatment [5], and protective device design [6, 7].

Limited quantitative datasets exist regarding the mechanical characteristics of soft tissues, and there is a scarcity of information concerning the mechanical behavior of soft tissues connected with human organs. Ethical considerations frequently impose constraints on the use of human models in biomedical and biomechanical research. There are great differences in the mechanical behavior of tissues from different species. To enhance our understanding of mechanical behavior in human organs, researchers often turn to animal models. Pigs, characterized by their physiological and anatomical resemblance to humans, particularly in the kidney and liver domains, stand out. The structural, dimensional, and vascular similarities between pig kidneys and livers and their human counterparts establish them as a preferred choice for emulating human organs. Moreover, the accessibility of pig organs facilitates experimental procedures.

There exists some literature on biomechanical and biological testing methods (Vincent [8]), which provides some information on sample fixation, uniaxial, biaxial or triaxial loading and testing in physiological media. However, to our knowledge,currently no testing standard suitable for the biological tissues exist. The existing literature includes some data on the mechanical properties of kidney, those experiments mainly are performed on cadavers and animals, and gives some information regarding uniaxial, biaxial, triaxial, shear loading, or impact loading on the physiological medium, besides conducting simulations of biomechanical response of the kidney. For example, Melvin et al. [9] studied the failure thresholds of maximum stress, maximum strain, and strain energy density (SED) of the whole-infused rhesus monkey kidney at different impact velocities. Farshad et al. [10] measured the mechanical properties of the porcine kidney in tension, compression, and shear manner. Karimi and Shojaei [11] first systematically studied the mechanical behavior of kidney human cadavers under the axial and transversal loadings. Umale et al. [12] measured the mechanical behavior of the porcine kidney cortex and capsule assuming the kidney is an isotropic material. Further, another study measured both the human and porcine kidney capsular membranes at different strain rates. It should be noted that the above-mentioned references belong to ex-vivo tests. Nava et al. [13] proposed an aspiration experimental approach to quantitatively in vivo measure the mechanical properties of the human kidney based on the study by Kauer [14] and Vuskovic [15]. The aspiration process recorded by the camera was used to obtain the mechanical properties of the kidney by fitting the finite simulation result with the experimental one. Although the aspiration test is proper for the in-vivo scene, the in vivo mechanical data may be affected by the blood flow and perfusion of internal organs [16, 17]. From above, it is obvious that there is a scarcity of information on the mechanical properties of the human kidney. As Mattei and Ahluwalia [18] discussed, although structural materials have been well characterized for decades using various testing methods,there remains a persistent lack of reliable and reproducible data for highly hydrated and degradable soft materials, such as hydrogels, and non-load-bearing biological tissues like the liver, kidney, and brain. This deficiency primarily stems from testing challenges associated with their shape, flexibility, and unstable properties. Typically, these materials exhibit a biphasic nature, comprising a solid network fully expanded within a surrounding liquid medium. Besides, the absence of a standardized testing protocol for assessing soft biological tissues reflects the difficulty in conducting the corresponding mechanical tests, such as tension and compression tests on extremely soft, fluid-filled tissues like the kidney. These tests necessitate specialized considerations, experimental setups, and procedures that may be beyond those used in traditional mechanical testing. Nonetheless, it is imperative to advance experiments concerning kidneys and supplement corresponding test data to enhance our comprehension of their mechanical behavior.

Whether the existing mechanical data of porcine renal tissues are similar and whether the mechanical data of porcine renal tissues are similar to the human kidney are largely unknown. Besides, the variability in test data renders these results ambiguous. Therefore, researchers still need to make experimental efforts to enrich the experimental mechanical database of Biomechanics. To this end, a baseline series of tension and compression experiments were performed to provide a complementarity to the existing dataset.

The existing mechanical constitutive models referring to the kidney mainly include a hyper-elastic constitutive equation [19], a hyper-viscoelastic model based on the concept of the strain energy function (e.g. Miller and Chinzei et al. [20, 21]; Miller [22, 23]; Umale et al. [12]), quasi-linear viscoelastic model [13]. All their model can properly characterize their experimental results. In conclusion, the existing mechanical constitutive models exhibit enough flexibility to describe the kidney tissue with the main characteristic of viscoelasticity or hyper-elasticity. However, the utilization of constitutive models may require a reasonable estimation of the model parameters in certain instances, as noted by Fazekas and Goda [24, 25]. Although phenomenological approaches demonstrate efficacy in predicting stress-strain responses under intricate loading conditions, employing constitutive models may demand additional efforts for parameter calibration in some cases, according to Fazekas and Goda [24, 25]. Moreover, thoes approach disregard progressing damage in the kidney during the loading, thus hardly reflect the damage evolution during the loading.

In this paper, the mechanical pressure-volume relation and viscoelastic material behavior of the porcine kidney were studied. To this end, the tri-compression test and strain rate-dependent tension test with a strain rate up to 0.1/s were performed. Based on the data gathered in this investigation, damage-dependent constitutive equations developed by us [26, 27] are suggested to model the mechanical behavior of the porcine kidney, which can be applied to the numerical simulation for informing the stress-strain behavior under certain loading cases.

## 2. Experimental method

Porcine kidney samples quickly extracted after the slaughter of the pig. Pig kidneys do not require ethics committee approval because the organs are obtained from local butchers and are provided for the local market. To ensure a wide diversity of data, eight pigs were selected from the ecologically bred pigs. The pigs are about 10 months old and weigh between 260 and 300kg. To prevent tissue dehydration, the organs were stored in a saline solution until tests were carried out. All the specimens were prepared and tested within 6 h after the death of the pig The preparation and test for all the specimens were performed within 6 h after the porcine death;. The test was conducted at room temperature (27°C) and 40% relative humidity. The samples to be tested were taken out from the saline solution, and the excess saline was tgently removed with absorbenttissues. This operation took about 3 min, after which the samples were tested immediately.

For each test condition, at least 5 samples were used. For some test conditions, up to 10 samples are prepared and tested. The choice to increase the number of samples was made based on the dispersion and reproducibility of the data.

**Fig 1:**
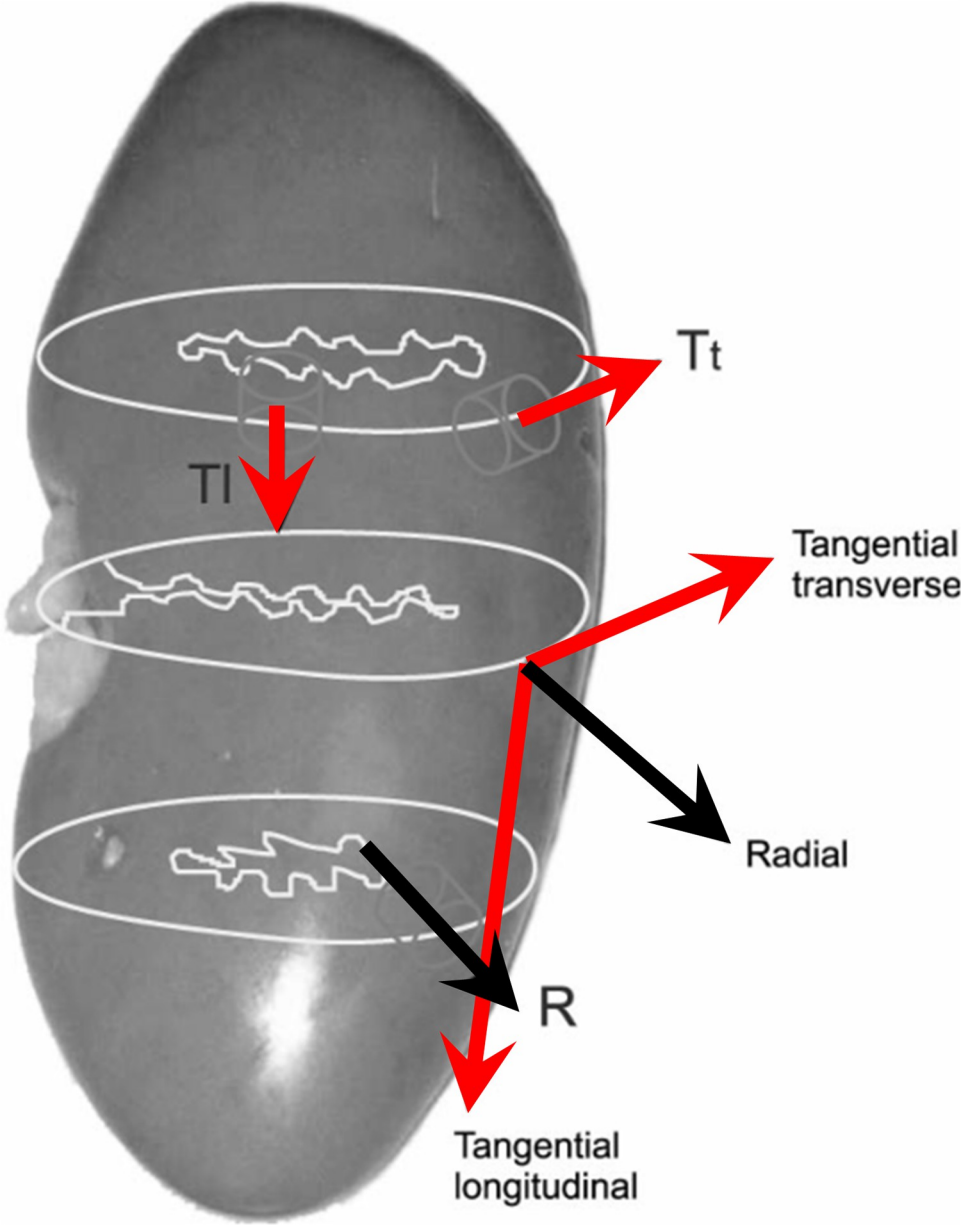
Schematic of the ‘‘radial” and the ‘‘tangential transverse” and ‘‘tangential longitudinal” directions in the kidney [10].

Roan and Vemaganti [28] mentioned that tissue from most cellular abdominal organs, such as the kidney and the liver, is considered to be isotropic, (visco-)hyperelastic, and incompressible. Research shows that the kidney manifests the globally inhomogeneous and also locally anisotropic [10, 29]. Fig 1 shows the geometrical form of the kidney [10], two different directions were used to characterize the material samples; one is the radial direction parallel with the blood vessel and the other is the tangential direction in line with the outer contour of the kidney. To be more precise, in this paper, all the samples were acquired from the outer capsule of the porcine kidney along the tangential longitudinal direction, besides, the capsule covering the cortex of the kidney was removed to avoid its disturbance to the experimental results because the existing study (Snedeker et al. [30]; Umale et al. [12]) shows that the capsule expresses a larger tensile strength compared with that of the outer cortex of the kidney.

### 2.1 Triaxial compression tests

**Fig 2:**
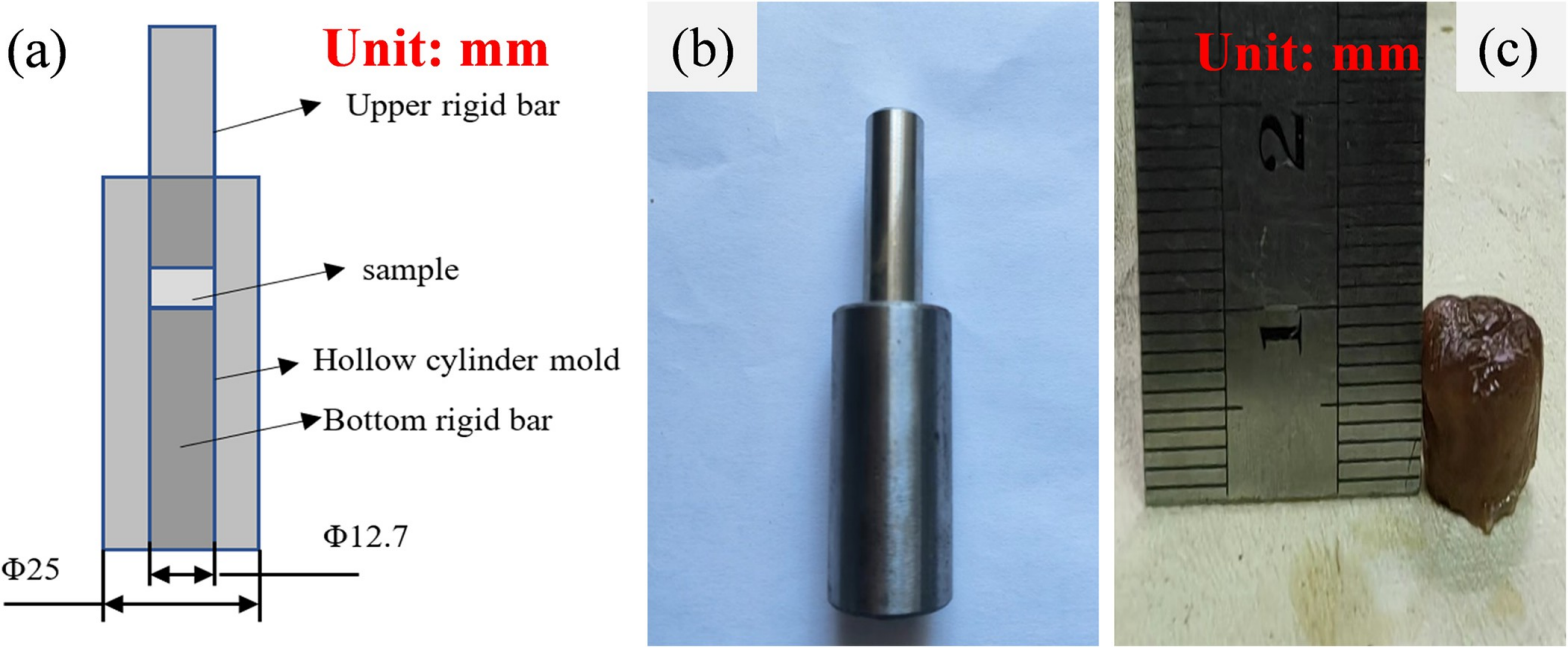
The triaxial compression tests. (a) shows the schematic diagram of compression chamber. (b) presents the real set-up of compression chamber. (c) presents the cylinder sample with a radius of 12.6 mm and a height of 8.0 mm.

Triaxial compression tests were carried out on a compression chamber consisting of a steel tube and a steel piston. Fig 2 schematically shows the test device for the triaxial compression tests. The test set was placed on the testing machine through which the load was applied uniaxially on the top of the piston. The triaxial compression tests of the porcine kidney were performed at the loading rate of 1mm/min corresponding to a strain rate of ~0.001/s in a static manner. The sample was prepared as a cylinder shape with a radius of 12.6 mm and a height of 8.0 mm, as shown in Fig 2C.

### 2.2 Uniaxial tension tests

**Fig 3:**
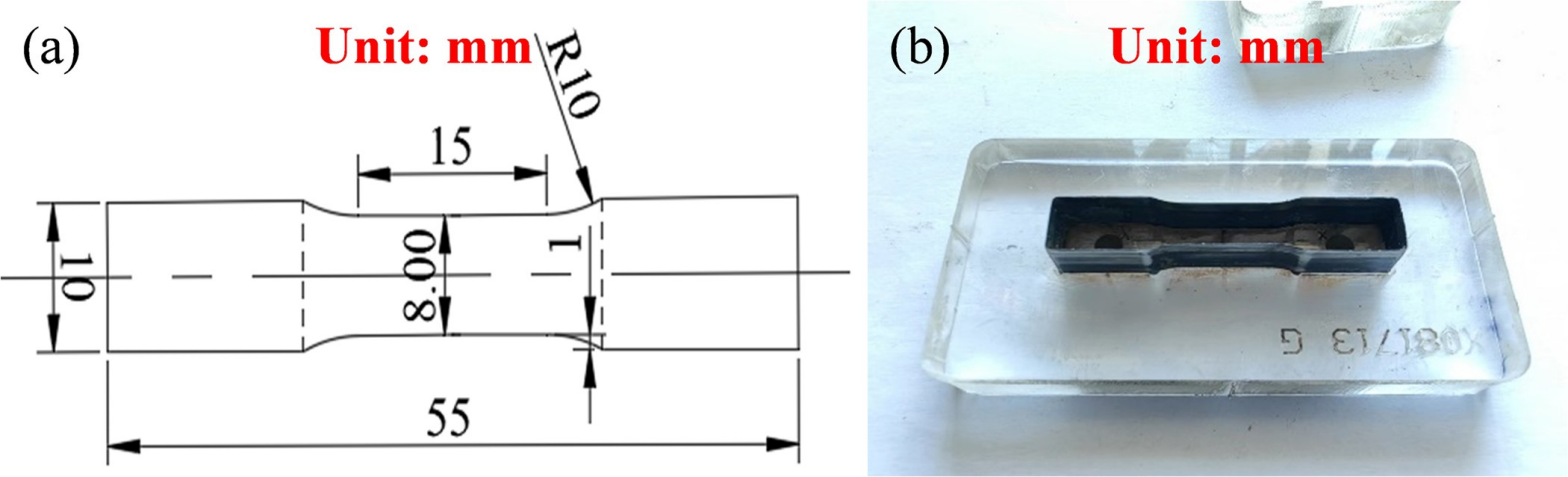
The mold blade used for uniaxial tension tests. (a) presents the schematic diagram of mold blade. (b) presents the real set-up of mold blade.

**Fig 4:**
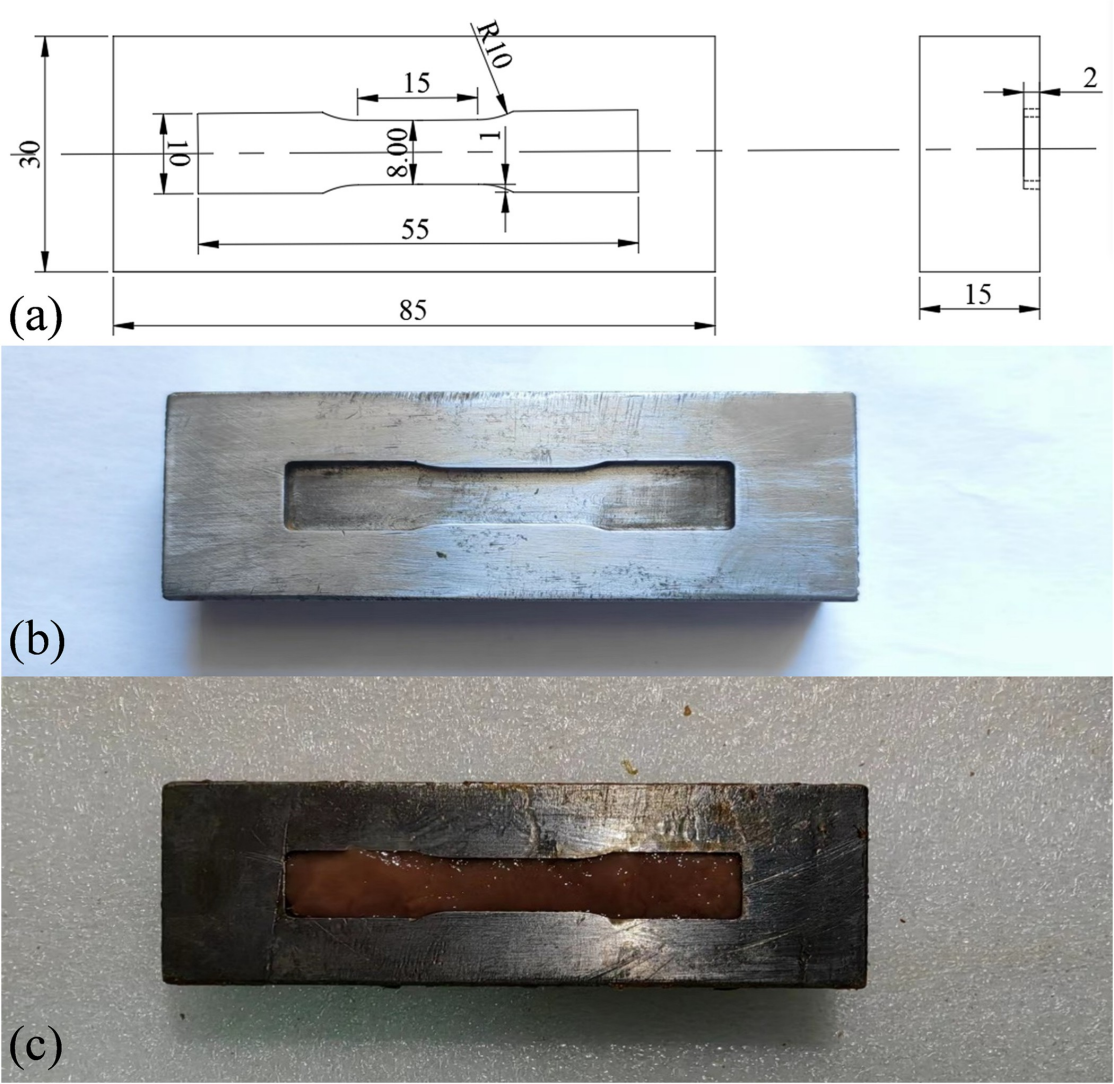
The mold used for uniaxial tension tests. (a) presents the schematic diagram of mold. (b) presents the real set-up of mold. (c) presents the final specimen in mold.

To perform tensile tests on the cortex, flat dog-bone-shaped specimens were prepared through the mold blade. The dimensions of the mold blade and its appearance are shown in Fig 3. First, the mold blade was used to cut the initial specimen from the kidney cortex with a thickness of about 4 mm. Then, this acquired specimen was placed on the specimen mold. The dimensions of the mold and its appearance are shown in Fig 4. By using this specimen mold, the specimen was made by the scalpel with final dimensions the same as that of the specimen mold, as shown in Fig 4C. All specimens were mounted on the testing machine by soft material clamp with saw-faced grips. The loading rates were set as 1mm/min, 10 mm/min and 100 mm/min corresponding to the strain rates of ~0.001/s, ~0.01/s and ~0.1/s. The displacement of the sample clamp region was measured by a high-speed camera. The area outside the dotted line is the clamping area as shown in Fig 3. Force data was recorded with a piezoelectric sensor with a maximum load of 500 N mounted in the universal testing machine.

### 2.3 Relaxation tests

In the relaxation tests, the preparation of the samples and their dimensions were the same as that in the uniaxial tensile test. The relaxation tests were performed with an initial displacement of 3.0 mm and a relaxation time of 20 minutes. The test results show that the time employed is long enough to achieve a stable force.

## 3. Experimental results and the calibration of constitutive equation

### 3.1 Bulk response of the porcine kidney

**Fig 5:**
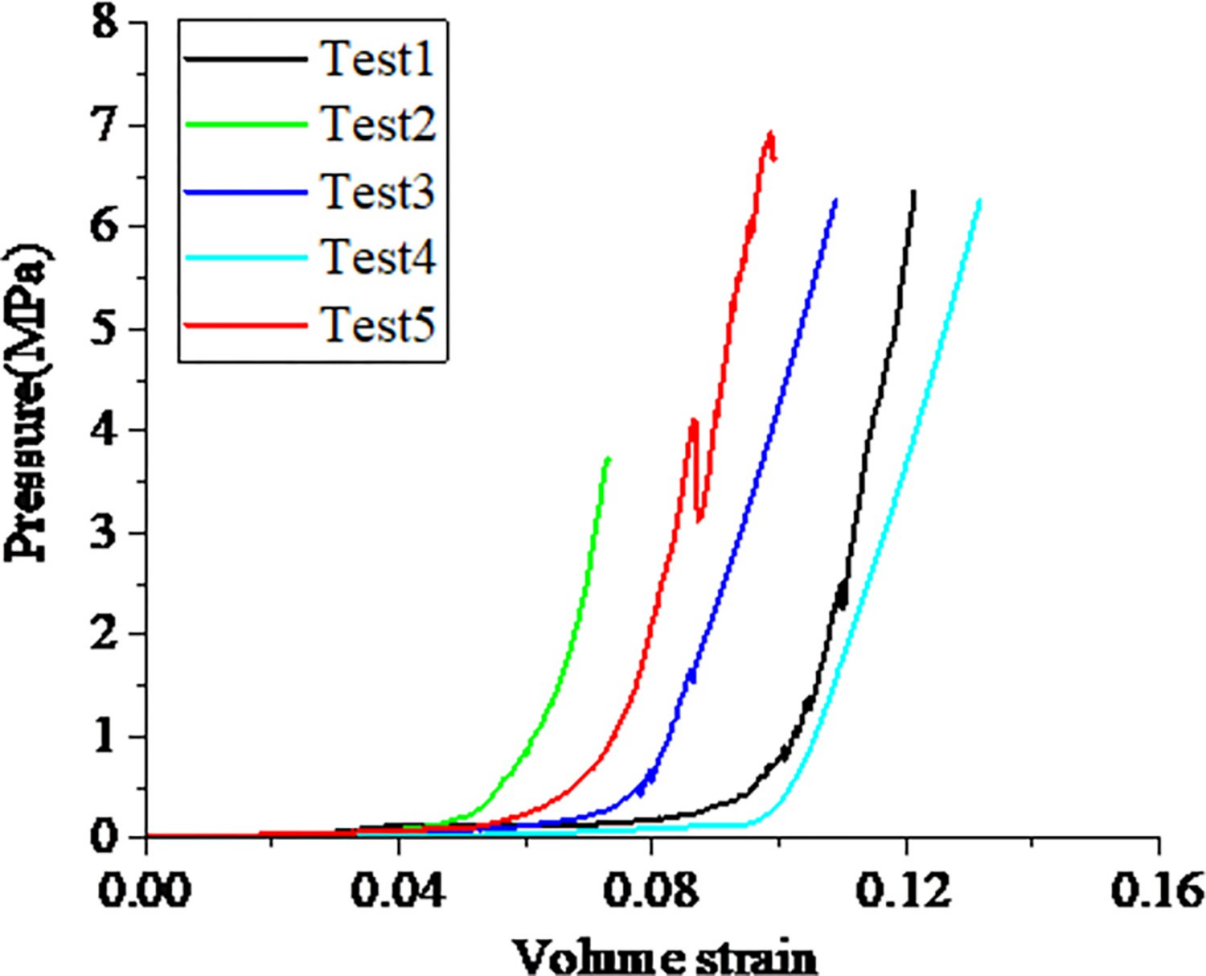
Pressure-volumetric strain data gained from the triaxial compression test on porcine kidney cortex.

Fig 5 shows the results of the triaxial compression tests on the porcine kidney cortex; it was displayed in the pressure versus volume strain coordinates. It can be seen that all test curves have two stages, in the first stage, the pressure varies slowly with the volumetric deformation; in the second stage, the pressure begins to rapidly increase with the volumetric deformation. Obviously, the durations of the first stage for different tests have some differences, besides, the trends of the second stage for different tests are similar but the slopes have some differences. During the triaxial compression tests, it was found that some tissue fluid seeps out of the mold in the first stage. Therefore, one reason for the disparity of duration in the first stage is the moisture of the sample. Besides, the texture of fiber for different samples may also contribute to this disparity. Further, the texture of fiber for different samples could result in a certain difference in modulus, thus causing certain differences in slopes for different tests in the second stage.

### 3.2 Tension response of the porcine kidney

**Fig 6:**
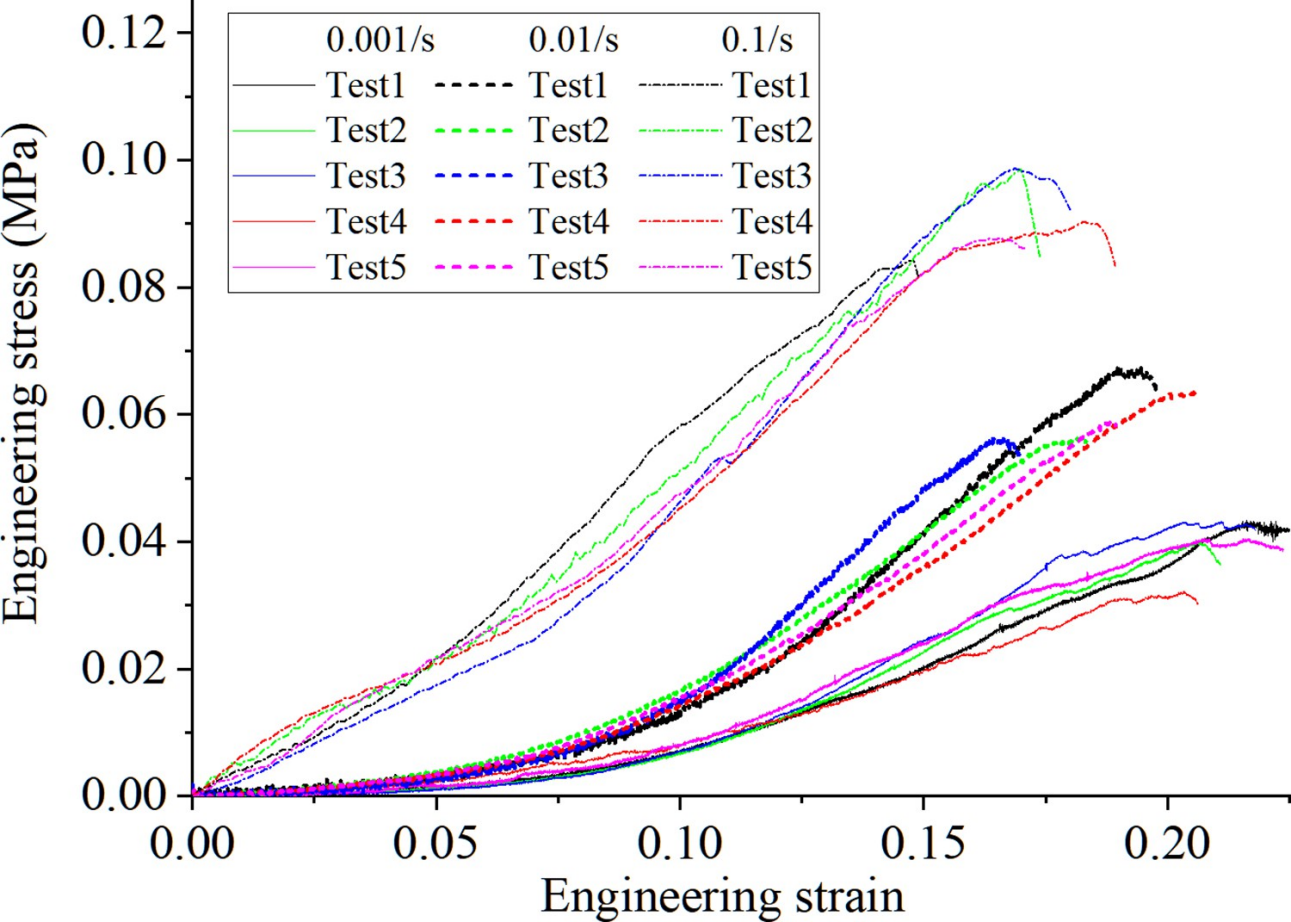
Strain rate effects on the porcine kidney cortex under uniaxial tension test with different loading strains.

Fig 6 presents the engineering stress-strain response of the cortex under different loading speeds in the uniaxial tension tests. Although an obvious disparity of the data occurs, a clear trend that a higher strain rate results in a higher ultimate strength but a lower rupture strain is concluded. Specifically, for the strain rate of 0.001/s, 0.01/s and 0.1/s, the ultimate engineering rupture stress are 0.0395MPa, 0.0599MPa and 0.0914MPa, and the engineering rupture strains are 0.218, 0.189 and 0.172, respectively. Besides, the initial Young modulus of the kidney cortex becomes larger with higher strain rates.

### 3.3 Relaxation tests

**Fig 7:**
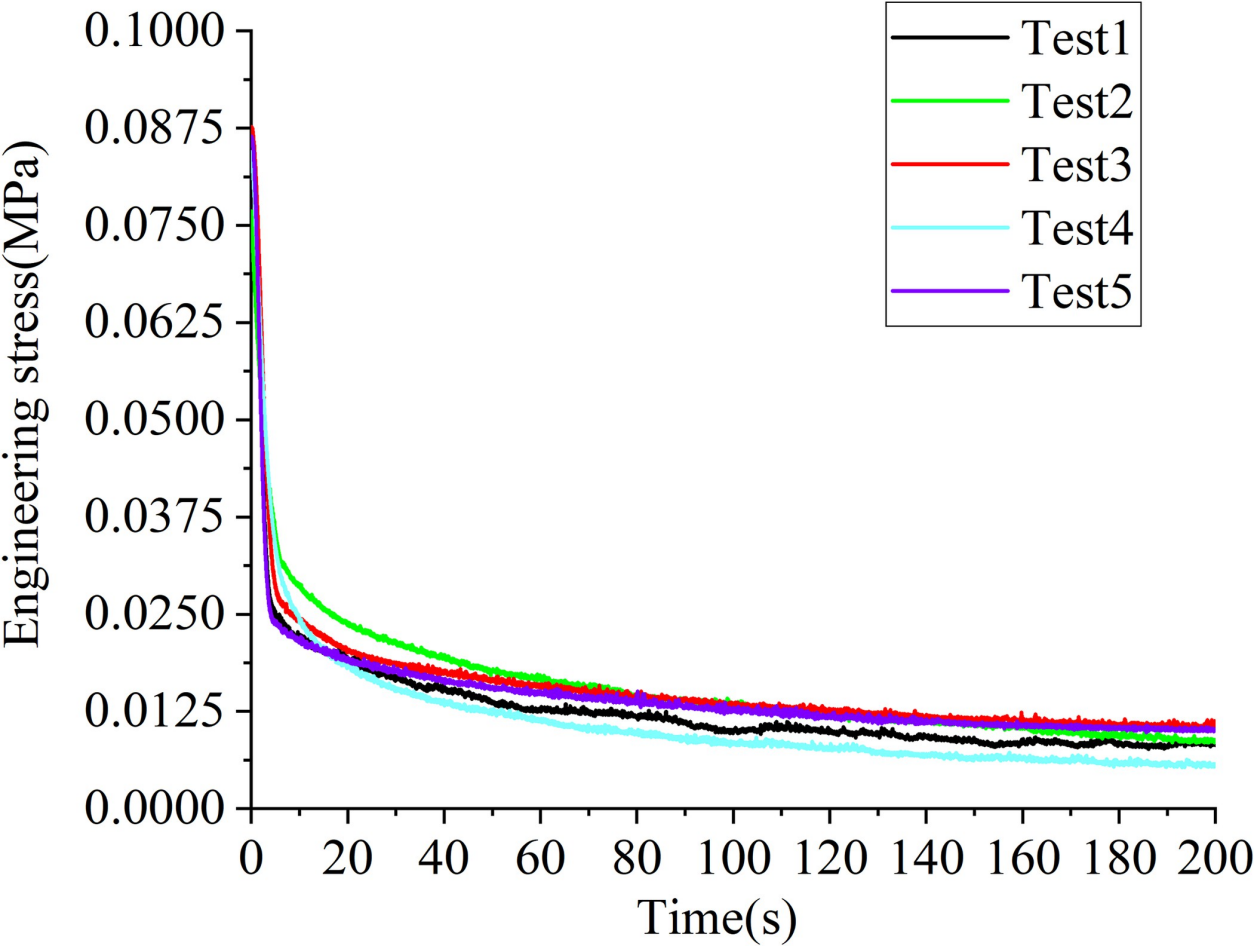
Engineering stress verse time data obtained from relaxation test on the porcine kidney cortex.

Fig 7 displays the results of relaxation tests in the form of engineering stress versus time. The stress rapidly drops with time in the initial 5s~10s duration, and then slowly drops with time. When the time arrives at the 200s, the stress nearly remains constant.

### 3.4 Constitutive model and calibration

As the experimental observations suggested, the material shows obvious viscoelasticity and volume change during loading. The damage-dependent visco-elastic consititutive equations [26, 27] are employed to model the mechanical behavior of the kidney cortex in tension at different strain rates and relaxation tests. The innovation of this model is that the progressing damage causing the stress degradation of the kidney cortex during the loading is considered.

In this model, the elastic response of the material is expressed as an additive decomposition of deviatoric and volumetric parts of strain energy density as:

$$\left\{ \begin{array}{l} W=\varphi (c)\cdot \omega ({\bar{I}}_{1},{\bar{I}}_{2})+\varpi (J)\\ \omega =C_{10}({\bar{I}}_{1}-3)+C_{20}{\left({\bar{I}}_{1}-3\right)}^{2}+C_{30}{\left({\bar{I}}_{1}-3\right)}^{3}\\ \varpi =\frac{1}{2}K{\left(J_{e}-1\right)}^{2} \end{array}\right.$$ (1)

where ${\bar{I}}_{1},{\bar{I}}_{2}$ denote the invariants of the right Cauchy-Green deformation tensor $\bar{C}=J^{-2/3}C$. *C*_10_, *C*_20,_ *C*_30_ are material constants, with which the intial shear modulus *G*_*0*_ is obtained as $2\cdot (C_{10}+C_{20}+C_{30})$. *K* is the bulk modulus. It should be noted that the shear modulus *G* and bulk modulus *K* in this model is changing with time. *c* represents the volume change due to damage (or, fluid leakage) and the volume ratio *J* = *J*_*e*_(1+*c*). *φ*(*c*) representsthe effect of damage on the distortional response and takes the following form,

φ(c)=1+ln(1−c) (2)

The volumetric and deviatoric elastic stresses are calculated from Eq (1),and expressed respectively as:

$$\bar{P}=\frac{\partial \varpi }{\partial J}\;\mathrm{and}\;S_{e}=\varphi (c)\cdot J^{-2/3}DEV(\frac{\partial \omega }{\partial \bar{E}})$$ (3)

where $\bar{E}=\frac{1}{2}(\bar{C}-I)$ and $DEV(•)=(•)-\frac{1}{3}[C:(•)]C^{-1}$.

Assuming that the shear and bulk relaxation of the material takes the same form, we obtain the viscoelastic response using convolution integrals on the elastic stresses,

$$P=\int_{0}^{t}m(t-\xi )\frac{\partial \bar{P}}{\partial \xi }d\xi \;\mathrm{and}\;S=\int_{0}^{t}m(t-\xi )\frac{\partial S_{e}}{\partial \xi }d\xi$$ (4)

with the dimensionless relaxation function

$$m(t)=m_{\infty }+\sum_{i=1}^{n}m_{i}e^{-t/\tau_{i}}$$ (5)

The shear modulus *G* can be gained by multiplying Eq (5) with initial shear modulus *G*_0_ as:

$$G(t)=G_{\infty }+\sum_{i=1}^{n}G_{i}e^{-t/\tau_{i}}$$ (6)

where *m*_*i*_ = *E*_*i*_/*G*_0_, *m*_∞_ = *E*_∞_/*G*_0_ and $G_{0}=E(0)=G_{\infty }+\sum_{i=1}^{n}G_{i}$. From Eq (6), the elastic modulus *E* and bulk modulus *K* can be transformed from shear modulus as:

E=2(1+μ)G (7)

$$K=\frac{2G(1+\mu )}{3(1-2\mu )}$$ (8)

where *μ* is assumed as 0.495, which means the incompressibility of tissue in the initial state.

Next, the growth rate of void volume fraction due to the damage in renal cortex is assumed to depend on the rate of distortion $\dot{\gamma }$ and substantially decreases with superimposed compressive pressure. Thus, the volume change form due to damage can refer to Özüpek and Becker [31]:

$$\dot{c}(t)=\dot{\gamma }e^{\alpha P},\;\gamma (t)=\beta {{\bar{I}}_{\gamma }}^{n}$$ (9)

where *α*, *β*, and *n* are constants and the octahedral shear strain ${\bar{I}}_{\gamma }$ is given as:

$${\bar{I}}_{\gamma }(t)=\frac{1}{6}\sqrt{2{\bar{I}}_{1}^{2}-6{\bar{I}}_{2}}$$ (10)

**Fig 8:**
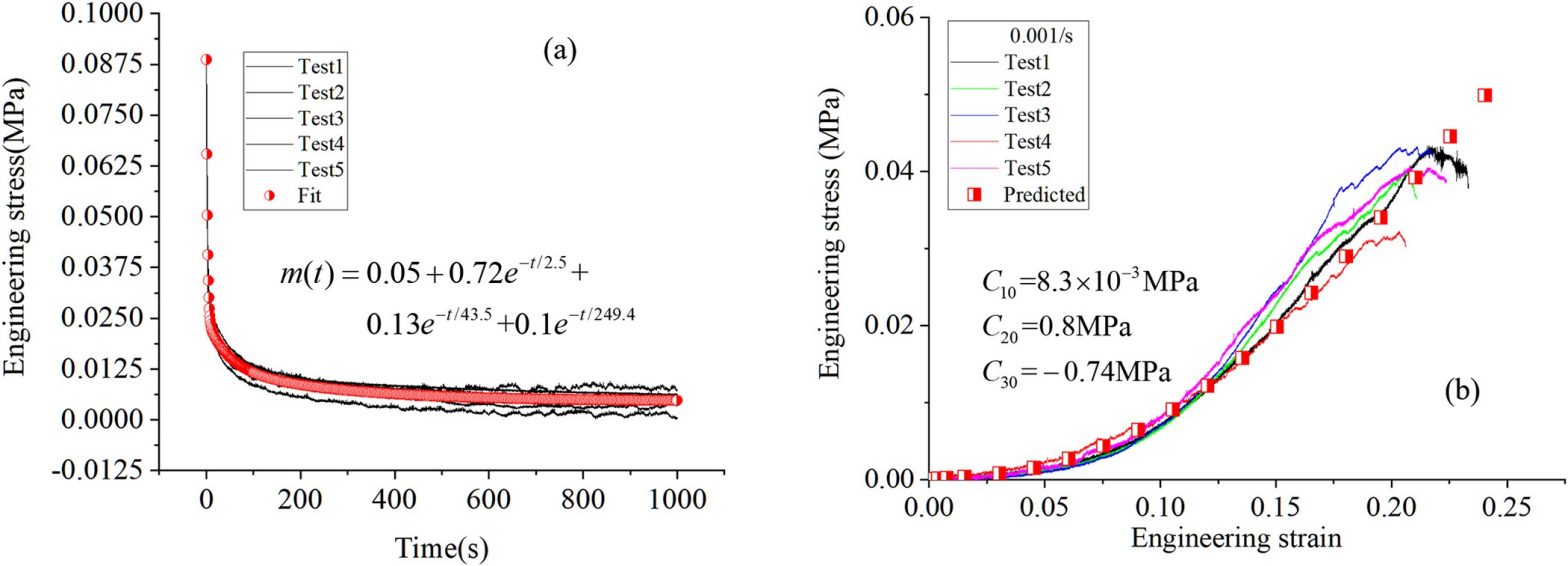
Determination of the model parameters. (a) dimensionless relaxation function fit. (b) calibration to the uniaxial tensile test data at 0.001/s.

Turning attention to the calibration process, the relaxation function in Eq 5 can be obtained by fitting the relaxation test data, and the results are shown in Fig 8A. The coefficients *C*_10_, *C*_20,_ *C*_30_ and *α*, *β*, and *n*. (Eq 6) was calibrated using the least squares technique for a uniaxial constant strain rate test at 0.001/s and room temperature, and the results are displayed in Fig 8B. The value of the initial bulk modulus is not important as long as it is chosen so that the material behaves as incompressible before damage begins.

**Fig 9:**
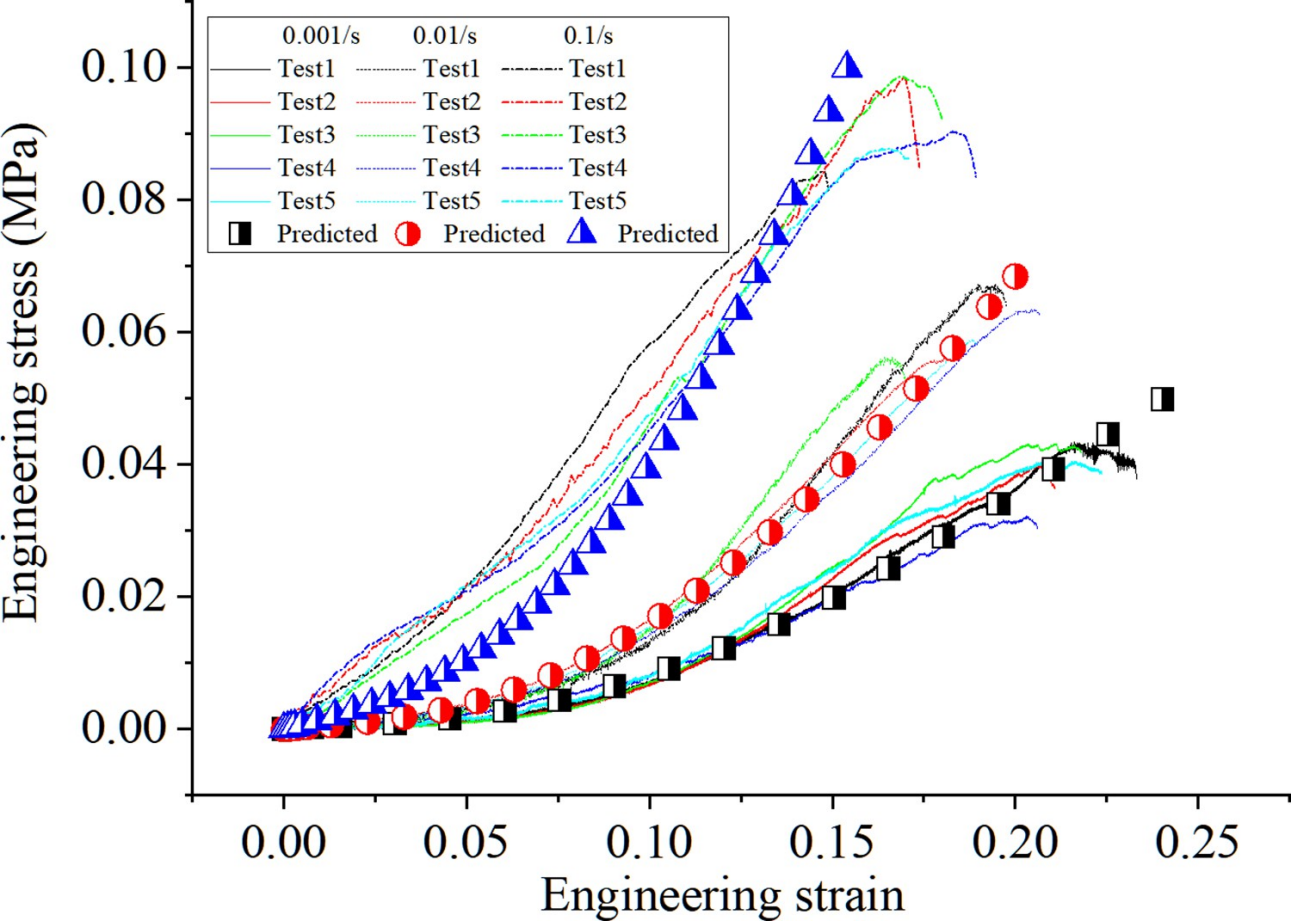
Experimental and predicted results under different strain rates.

After determining the damage function, the current model was imported into ABAQUS software for numerical prediction, and the model parameters were shown in Table 1. The model prediction under uniaxial tension is shown in Fig 9, where the results agree well with the measured ones, indicating that the proposed model can predict the mechanical behavior of the material. Unfortunately, due to the lack of failure criteria, the current model cannot predict the elongation at break.

**Table 1:** Model parameters for the studied material.

*C*_10_ (MPa)	*C*_20_ (MPa)	*C*_30_ (MPa)	*β*	*n*
8.3e-3	0.8	-0.74	3.0e-4	2.0

It is noteworthy that the initial stage prediction of 0.1/s shows a slight deviation from the experimental results, which may be attributed to the variability in tissue fluid content within the samples and the subsequent seepage at different flow rates during the test. Besides, the test pressure-volume data is not modeled by our constitutive model, this is because some liquid leaks out during the tri-compression test, which may need a fluid-solid coupling approach to reproduce the corresponding test data, which is our future research point.

## 4. Discussion

This paper presents the data of the triaxial compression test, uniaxial tension of different strain rates, and relaxation test on the porcine kidney cortex. Those test results are an important complement to the current mechanical data set of living tissues, which is necessary to better understand the behavior of the porcine kidney cortex. The uniaxial tension data of the porcine kidney cortex in this paper is compared with that by Karimi and Shojaei [11], both at a test strain rate of about 0.001/s. Karimi and Shojaei [11] reported that the average ultimate strength of the human kidney cortex is 0.0285MPa and the rupture strain is about 11.5%, and our test data of the porcine cortex with an average ultimate strength of 0.0395MPa and a rupture strain about 21.8%. It seems that the porcine kidney has better strength and ductility than that of the human kidney, but the difference between them is minor. The uniaxial test data in this paper is also compared with the data from Farshad et al. [10] at the strain rate of 0.001/s, both test data are about the porcine kidney cortex, but the former is obtained from the uniaxial tension test with an average ultimate strength of 0.0395 MPa, and rupture strain about 21.8%, the latter is from uniaxial compression test with an average ultimate strength of 0.18 MPa and rupture strain about 47%. It appears that the strength and ductility of the porcine kidney cortex in compression are better than that in tension, which can be further illustrated by comparing with the uniaxial compression data by Umale et al. [12], who reported that the average ultimate strength is about 0.13 MPa and the rupture strain is about 37%. From above, it seems that the mechanical behavior of the porcine kidney cortex manifests an obvious stress state-dependent mechanical behavior.

The test data of mechanical behaviors in porcine kidney cortex samples exhibit a certain scatter, this may attributed to the difference in the texture of fiber and the uneven thickness of the prepared samples. Another factor causing the scatter of data may attributed to the specimen preparation process and the challenges associated with consistently maintaining uniform moisture conditions for the samples throughout their extraction, preparation, and during the actual tests. Although the test data manifests a certain scatter, the whole trend or regular is clear. Specifically, for the triaxial compression test, the pressure-volume relation of different tests displays some difference in the initial compression stage but exhibits the same trend when the kidney cortex is compressed to a certain extent. For the uniaxial tension test, the kidney shows an obvious strain rate effect on the stress-strain relation. With the higher strain rate, the ultimate strength becomes larger but the rupture strain is lower. And these results have the same trend as that obtained in the uniaxial compression test by Farshad et al. [10].

By using our recently proposed visco-elastic model, the tension data at different strain rates and relaxation data can be modeled accurately. The advantage of this model is that the calibration of the corresponding parameter is easy and the progressing damage in the kidney cortex during the loading is considered. The good agreement between the predicted results and the test data demonstrates that the damage has an obvious influence on the stress-strain relation.

Several limitations still exist in this study, which need to be further conducted in future studies. (1) Due to the lack of failure criteria, the current constitutive model cannot predict the elongation at break. (2) The triaxial compression is only performed on the strain rate at 0.001/s corresponding to the static manner, next step, more higher strain rate tests need to be done to present the strain rate-dependent mechanical behavior of the kidney pressure-volume relation.

## 5.Conclusion

With the development of medical disease diagnosis, virtual reality simulation, trauma medical treatment, and protective device design, knowing the mechanical properties of living tissue become increasingly important. The scarcity of information about the mechanical properties of the kidney requires further study in this field. This paper performed the mechanical test on the porcine kidney cortex and acquired the pressure-volume relation and viscoelastic material behavior of the porcine kidney. Then, our proposed damage-dependent constitutive model was employed to fit the experimental results, which is beneficial for computer simulation. The current study is not only for further understanding the mechanical properties of the kidney but also for medical and biomechanical purposes to be used for diagnosis and simulations, respectively.

## Supporting information

S1 Data (XLSX)
