# Supplementary material for: Evaluation of the mechanical properties of porcine kidney
Source: PLoS One. 2024 Jul 25;19(7):e0307778. doi: 10.1371/journal.pone.0307778 (PMC11271945; doi:10.1371/journal.pone.0307778)
Supplement: Attachment — Submitted filename: Response to Reviewers.docx [file pone.0307778.s002.docx]

Dear Editors and Reviewers:

Thank you for your letter and for the reviewers’ comments concerning our manuscript entitled “Evaluation of the mechanical properties of porcine kidney” (Manuscript Number: PONE-D-24-00404R1). Those comments are all valuable and very helpful for revising and improving our paper, as well as the important guiding significance to our researches. We have studied comments carefully and have made correction which we hope meet with approval. Revised portion are marked in bold and blue fonts in the revised manuscript. The main corrections in the paper and the responds to the reviewer’s comments are as flowing:

Responds to the Editors and reviewer’s comments:

Journal requirements:

1. Please ensure that your manuscript meets PLOS ONE's style requirements, including those for file naming.

Reply: Thanks for your comments.

We did a further check to ensure that our manuscript meets PLOS ONE's style requirements, including those for file naming.

2. We noticed you have some minor occurrence of overlapping text with the following previous publication(s), which needs to be addressed:

- Material characterization of the pig kidney in relation with the biomechanical analysis of renal trauma - https://doi.org/10.1016/S0021-9290(98)00180-8

- Sample, testing and analysis variables affecting liver mechanical properties: A review - https://doi.org/10.1016/j.actbio.2016.08.055

- A unified viscoelastic model of progressive damage and failure for solid propellants - https://doi.org/10.1016/j.ijplas.2023.103765

- Multiscale viscoelastic constitutive modeling of solid propellants subjected to large deformation - https://doi.org/10.1016/j.ijsolstr.2022.112084

- Constitutive modeling of solid propellants for three dimensional nonlinear finite element analysis - https://doi.org/10.1016/j.ast.2017.06.025

In your revision ensure you cite all your sources (including your own works), and quote or rephrase any duplicated text outside the methods section. Further consideration is dependent on these concerns being addressed.

Reply: Thanks for your comments. That is a good suggestion! This manuscript was made a similarity examination. The sentences with high similarity with exsiting references were modified to reduce the overlaps.

3. Please note that PLOS ONE has specific guidelines on code sharing for submissions in which author-generated code underpins the findings in the manuscript. In these cases, all author-generated code must be made available without restrictions upon publication of the work. Please review our guidelines at https://journals.plos.org/plosone/s/materials-and-software-sharing#loc-sharing-code and ensure that your code is shared in a way that follows best practice and facilitates reproducibility and reuse.

Reply: Thanks for your comments.

All codes cannot be shared publicly. The constitutive model writing involved in this manuscript is the author's own achievement and intellectual property of the author. But the generic model is publicly shareable. In addition, we are responsible for ensuring that the code is reusable and well documented.

4. Thank you for stating the following financial disclosure:

“The authors would like to thank China National Nature Science Foundation (Grant no. 11872119).”

Please state what role the funders took in the study. If the funders had no role, please state: "The funders had no role in study design, data collection and analysis, decision to publish, or preparation of the manuscript."

If this statement is not correct you must amend it as needed.

Please include this amended Role of Funder statement in your cover letter; we will change the online submission form on your behalf.

Reply: Thanks for your comments.

We further state what role the funders took in the study as follows. At the same time, we have added descriptions to the revised manuscript.

"The funders had no role in study design, data collection and analysis, decision to publish, or preparation of the manuscript. However, The funders provide financial help in polishing the manuscript."

5. When completing the data availability statement of the submission form, you indicated that you will make your data available on acceptance. We strongly recommend all authors decide on a data sharing plan before acceptance, as the process can be lengthy and hold up publication timelines. Please note that, though access restrictions are acceptable now, your entire data will need to be made freely accessible if your manuscript is accepted for publication. This policy applies to all data except where public deposition would breach compliance with the protocol approved by your research ethics board. If you are unable to adhere to our open data policy, please kindly revise your statement to explain your reasoning and we will seek the editor's input on an exemption. Please be assured that, once you have provided your new statement, the assessment of your exemption will not hold up the peer review process.

Reply: Thanks for your comments.

All the data in this manuscript are true and reliable. We can provide all the data after the manuscript is accepted when the journal needs it.

6. PLOS requires an ORCID iD for the corresponding author in Editorial Manager on papers submitted after December 6th, 2016. Please ensure that you have an ORCID iD and that it is validated in Editorial Manager. To do this, go to ‘Update my Information’ (in the upper left-hand corner of the main menu), and click on the Fetch/Validate link next to the ORCID field. This will take you to the ORCID site and allow you to create a new iD or authenticate a pre-existing iD in Editorial Manager. Please see the following video for instructions on linking an ORCID iD to your Editorial Manager account: <https://www.youtube.com/watch?v=_xcclfuvtxQ>.

Reply: Thanks for your comments.

ORCID iD was provided by the corresponding author. It is also verified in the edit manager.

7. Please include a separate caption for each figure in your manuscript.

Reply: Thanks for your comments.

We placed the figures in the manuscript in a separate file, and clearly marked the position of the figures and tables in the original manuscript with bold and blue fonts.

Reviewer #1:

The paper titled "Evaluation of the Mechanical Properties of Porcine Kidney" by Zhao Zhang, Xianglong Tan*, Mengyang Li, Wubuliaisan Ma, Shangjian Zeng, and Yanqing Wu* investigates the mechanical properties of fresh porcine kidneys, focusing on their viscoelastic material behavior. I am having a hard time to figure out the novelty of this paper!

1. The term "fracture" for soft biological tissues does not seem accurate. Maybe rupture or tissue failure would be a better choice.

Reply: Thanks for your comments. That is a good suggestion!

We have used the term "rupture" to take the place of the term "fracture" in revised manuscript. Revised portion are marked in bold and blue fonts in the revised manuscript.

2. While the introduction well acknowledged the work that has been done so far, I am still confused what has been missing in the field that you try to address in this paper? just different strain rates? just different material models? I do not think all this is original enough for a publication!

Reply: Thanks for your comments.

A good understanding of the mechanical properties of living organs or issues are critical for many real applications, such as medical disease diagnosis [1,2], virtual reality simulation [3,4], trauma medical treatment [5], and protective device design [6,7]. Through the literature review, we found that the uniaxial tensile test of pig kidney under different strain rates has not been carried out, and the corresponding constitutive model construction work is still lacking. The work in this paper plays an important role in fully understanding the mechanical properties of pig kidney. The work in this manuscript may be useful for biological simulation, because finite element simulation requires an understanding of material behavior and a constitutive model. Besides, this work may contribute to the medical diagnosis of kidney, because the mechanical properties of the normal kidney and the diseased kidney are different.

3. What made you to choose such different strain rates? from 1 mm/min to 100 mm/s? you sure that is not a typo?

Reply: Thanks for your comments. That is a good suggestion!

Through a reexamination of the manuscript, the "1mm/s", "10mm/s" and "100mm/s" in section 2.2 are truly typos. Those content has been modified as "1mm/min", "10mm/min" and "100mm/min".

The chosen three loading rates correspond to strain rates of 0.001/s, 0.01/s, and 0.1/s, which are common in mechanical tests of soft tissues from static tests to slow dynamic tests. Revised portions are marked in bold and blue fonts in the revised manuscript.

4. Abaqus has all these material models and can calculate these properties so why did you add to this?

Reply: Thanks for your comments.

The constitutive model employed in this paper is our recently developed damage-dependent viscoelastic constitutive equations. The innovation of this model is that the progressing damage causing the degradation of the kidney cortex during the loading is considered. By using the existing models in Abaqus, the strength-reduced behavior in modeling different tests cannot be achieved. Thus, our recently constitutive model is coded into Abaqus, and can provide good agreement with the test data.

5. Why even we need to know about the "fracture" of this tissue? You do not have any information in the paper that why do we need to know about this?

Reply: Thanks for your comments. Through our uniaxial tension at different strain rates, it is found that the engineering rupture strains are 0.218, 0.189, and 0.172 for the strain rates of 0.001/s, 0.01/s, and 0.1/s, respectively. It is concluded that the rupture strain decreases with the increase of the strain rate. Due to the structural, dimensional, and vascular similarities between pig kidneys and human counterparts, the rupture strain information of pig kidney can serve different scenarios. For example, it can guide the operation of human kidney surgery to avoid excessive force. Besisde, it can giude the design of biological protective equipment under blunt impact.

6. Could not find any limitation section?

Reply: Thanks for your comments.

The shortcomings of the model are described in this paper, such as the lack of failure criteria and the inability of the current model to predict the elongation at break. The sentence in manuscript is ‘’Unfortunately, the current model cannot predict the elongation at breakage due to the lack of failure criterion.’’ Future work with consideration of failure criterion will be done to well describe the rupture behavior of porcine kidney.

Reviewer #2:

Zhang et al show an interesting study on physical characteristics of porcine kidney based on experimental data (on tension, compression and relaxation) supported by constitutive modeling. However, poor presented/substantiated methodology and findings make it difficult to understand and impair overall impact of the work. Below are few comments to guide authors.

1. Abstract too long and conclusions may be made concise

Reply: Thanks for your comments. That is a good suggestion!

The abstract part in the manuscript has been revised to make it more concise. Revised portion are marked in bold and blue fonts in the revised manuscript.

2. Triaxial compression tests, how such a loading rate was selected and it compares to physiological values??

Reply: Thanks for your comments. That is a good suggestion!

The loading speed chosen in triaxial compression corresponds to the case of quasi-static compression. The strain rate can be approximated by dividing the compressive loading speed by the sample height. The strain rate equal or below 0.001/s can be viewed as static manner, under such the mechanical behavior of the material tested can help inform the mechanical property test under slow loading. In this paper, the triaxial compression mechanical properties test is mainly done under quasi-static conditions, and the triaxial compression test is beneficial to understand the volume stress and volume strain relationship of materials under the pressure loading.

3. Uniaxial tension tests, why three strain rate values were selected and they compare to physiological values?

Reply: Thanks for your comments.

For the uniaxial tension test, three different loading rates were set as 1mm/min, 10mm/min and 100mm/min corresponding to the strain rates of ~0.001/s, ~0.01/s and ~0.1/s. This strain rate range corresponds to the material mechanical behavior under quasi-static loading to the slow impact loading. Thus, the test and the corresponding constitutive relation can help us inform the porcine behavior under such loading range. One application of this information is it can be used in simulating human damage under blunt impact to aid the protective equipment.

4. Relaxation tests: Why 20 mins and 3 mm (not less or more) for relaxation tests?

Reply: Thanks for your comments. That is a good suggestion!

Relaxation test is used to obtain the stress-time behavior under the constant displacement or strain, which can reflect the viscosity of the tested material. It should be noted that the time duration used in the test should ensure a stable force appear with the loading time, thus the relaxation character of the material can be revealed. We found an initial displacement of 3.0 mm and a relaxation time of 20 minutes is able to achieve the stable force.

5. A lot of raw data is shown in but the relation among results obtained by different techniques (including effect of strain rate), and statistical caparison is missing (maybe bar chart?)

Reply: Thanks for your comments.

In the revised paper, a chart was added to clearly provide the comparison of the different data. Then the discussion followed.

6. L197: Where did you calculate elastic modulus and which elastic modulus (E or K)?

Reply: Thanks for your comments. To illustrate how we get the elastic modulus and the relation between the elastic modulus *E* bulk modulus *K* and shear modulus *G*, some complementary content was added. This modification was presented in the revised manuscript. The modification is as below:

In this model, the elastic response of the material is expressed as an additive decomposition of deviatoric and volumetric parts of strain energy density as:

(1)

where $\bar{I}_{1},\bar{I}_{2}$ denote the invariants of the right Cauchy-Green deformation tensor $\bar{\mathbf{C}}=J^{-2/3}\mathbf{C}$. *C*_10_, *C*_20,_ *C*_30_ are material constants, with which the intial shear modulus *G_0_* is obtained as $2\cdot(C{}_{10}+C{}_{20}+C{}_{30})$. *K* is the bulk modulus. It should be noted that the shear modulus *G* and bulk modulus *K* in this model is changing with time.$c$represents the volume change due to damage (or, fluid leakage) and the volume ratio $J=J_{e}(1+c)$. representsthe effect of damage on the distortional response and takes the following form,

(2)

The volumetric and deviatoric elastic stresses are calculated from Equation (1) and expressed respectively as:

$\bar{P}=\frac{\partial\varpi}{\partial J}$ and(3)

where $\bar{\mathbf{E}}=\frac{1}{2}(\bar{\mathbf{C}}-\mathbf{I})$ and $DEV(\bullet)=(\bullet)-\frac{1}{3}[\mathbf{C}:(\bullet)]\mathbf{C}^{-1}$.

Assuming that the shear and bulk relaxation of the material takes the same form, we obtain the viscoelastic response using convolution integrals on the elastic stresses,

$P=\int_{0}^{\text{t}} m\left(t-\xi\right)\frac{\partial\bar{P}}{\partial\xi}\text{d}\xi\begin{matrix} , & \mathbf{S}=\int_{0}^{\text{t}} m\left(t-\xi\right)\frac{\partial\mathbf{S}_{e}}{\partial\xi}\text{d}\xi\end{matrix}$ (4)

with the dimensionless relaxation function

$m(t)=m_{\infty}+\sum_{i=1}^{n} m_{i}e^{-t/\tau_{i}}$ (5)

The shear modulus *G* can be gained by multiplying Eq.(5) with initial shear modulus *G*_0_ as:

$G(t)=G_{\infty}+\sum_{i=1}^{n} G_{i}e^{-t/\tau_{i}}$ (6)

where $m_{i}=E_{i}/G_{0}$ , $m_{\infty}=E_{\infty}/G_{0}$ and$G_{0}=E(0)=G_{\infty}+\sum_{i=1}^{n} G_{i}$. From Eq.(6), the elastic modulus *E* and bulk modulus *K* can be transformed from shear modulus as:

$E=2(1+\mu)G$ (7)

$K=\frac{2G(1+\mu)}{3(1-2\mu)}$ (8)

From above, the elastic moduls in this model is a variable that changes with time. The intial elastic moduls *E*_0_ is a constant and expressed as $E_{0}=E(0)=E_{\infty}+\sum_{i=1}^{n} E_{i}$. Thus, the elastic modulus defined in this paper is different from other research, more attention in this paper is on the prediction of the test stress-strain curves and the test index of ultimate strength and rupture strain.

7. L204: The justification for constitutive model selection is not provided, and physical significance of the selected parameters may be provided for better interpretation of the findings.

Reply: Thanks for your comments.

As the experimental observations suggested, the material shows obvious viscoelasticity and volume change during loading. Thus a viscoelasticity model is needed. The model used in this papar is a damage-dependent visco-elastic consititutive equations, which have three parameter *C*_10_, *C*_20,_ *C*_30_ controling the visco-elastic-stress variation trend and three parameter *α*, *β*, and *n* adujsting the effect of damage on the stress degradation of the kidney.

The model used in this paper exhibits good flexibility in predicting our test data. Besides, for other viscoelastic materials like propellent, and elastomers[], this model also exhibits a good performance in predicting the corresponding test data. It is believed that this model has good applicability to the viscoelastic mechanical behavior of other biological materials.

8. L266, L271,L272: Which strength?

Reply: Thanks for your comments.

"strength" at L264，L266，L271，L272，L276 in original manuscript is for "ultimate strength", the ultimate engineering stress and used " ultimate strength " to take the place of "strength". Revised portion are marked in bold and blue fonts in the revised manuscript.

9. Figs title may elaborate the fig to guide the reader.

Reply: Thanks for your comments.

Revised portion are marked in bold and blue fonts in the revised figure document.

10. Fig.1: Color contrast may be adjusted to improve contrast; Fig.2 & 3: units missing

Reply: Thanks for your comments. That is a good suggestion!

The color contrast of Fig.1 is supplemented in detail to make its distinction between tangential and radial directions more obvious. The missing units in Fig.2&3 are supplemented. Revised portion are marked in bold and blue fonts in the revised figure document.

Fig.1 Schematic of the ‘‘radial’’ and the two ‘‘tangential’’ directions in the kidney^[10]^

Fig.2 The triaxial compression tests (a)schematic (b) real set-up.

Fig.3 The mold blade used for uniaxial tension tests (a)schematic of dimension (b) real set-up.

11. Discussion fails to compare obtained modulus values to existing values in literature (e.g. ~ 1-10kPa range for Kidney). While authors obtain ~30kPa, such difference exists may be discussed…

Reply: Thanks for your comments.

12. Introduction may mention about the difference among tissues from different species….

Reply: Thanks for your comments.

This paper concerns the mechanical behavior of kidneys. Besides, the structural, dimensional, and vascular similarities between pig kidneys and human counterparts are high. The mechanical information about porcine kidneys is very useful to understand the human itself. Thus, in the Introdctuion and subsequent Disscussion, references about mechanical behaviors in human and porcine were given.

The content of the article has been modified as follows. Revised portion are marked in bold and blue fonts in the revised manuscript.
