# Supplementary material for: Evaluation of the mechanical properties of porcine kidney
Source: PLoS One. 2024 Jul 25;19(7):e0307778. doi: 10.1371/journal.pone.0307778 (PMC11271945; doi:10.1371/journal.pone.0307778)
Supplement: Attachment — Submitted filename: Review report_PONE-D-00404_R1.docx [file pone.0307778.s003.docx]

Since the revised version don’t seem to provide sufficient literature references indicating physiological values that has been used by authors as a basis for their experiments and the edits made by authors based on suggestions seem unclear in the revised manuscript version (e.g edits in response to each comment need to be clearly indicated by corresponding Line numbers in manuscript), I would seek a major revision. The below points are merely indicative, and the authors should carefully revise the manuscript to significantly improve the reader’s confidence.

1. Triaxial compression tests, how such a loading rate was selected and it compares to physiological values??

“The strain rate equal or below 0.001/s can be viewed as static manner, …”

Is it percent strain rate may be clarified?

2. Uniaxial tension tests, why three strain rate values were selected and they compare to physiological values?
3. Three three strain rates selected here vary by two orders of magnitude and still considered quasistatic by authors.
4. Material and Methods: Contains a paragraph (L137- L149) that seems more like introductory.
5. Limitations of the study may be clarified
