# Supplementary material for: Evaluation of the mechanical properties of porcine kidney
Source: PLoS One. 2024 Jul 25;19(7):e0307778. doi: 10.1371/journal.pone.0307778 (PMC11271945; doi:10.1371/journal.pone.0307778)
Supplement: Attachment — Submitted filename: Response to Reviewers.docx [file pone.0307778.s004.docx]

Manuscript Number：PONE-D-24-00404R1

**Title: Evaluation of the mechanical properties of porcine kidney**

Authors: Zhao Zhang, Xianglong Tan, Mengyang Li, Wubuliaisan, M, Shangjian Zeng, Yanqing Wu.

Corresponding author: tanxianglong301@163.com; wuyqing@bit.edu.cn

Response to Reviewer

General comments:

Since the revised version don’t seem to provide sufficient literature references indicating physiological values that has been used by authors as a basis for their experiments and the edits made by authors based on suggestions seem unclear in the revised manuscript version (e.g edits in response to each comment need to be clearly indicated by corresponding Line numbers in manuscript), I would seek a major revision. The below points are merely indicative, and the authors should carefully revise the manuscript to significantly improve the reader’s confidence.

**Reply:**

Thanks to the Reviewer for their comments concerning our manuscript entitled “Evaluation of the mechanical properties of porcine kidney” (Manuscript Number: PONE-D-24-00404R1). Those comments are all valuable and helpful for further improving our paper and as the important guiding significance to our researchers. We have studied comments carefully and have made correction which we hope meet with approval. Revised portion are marked in bold and blue fonts in the revised manuscript. The responses to the comments by the reviewer are as follows.

1. Triaxial compression tests, how such a loading rate was selected and it compares to physiological values? “The strain rate equal or below 0.001/s can be viewed as static manner, …” , Is it percent strain rate may be clarified?

Thanks for your comments. From the mechanical concept, the strain rate is the change in strain (deformation) of a material with respect to time, many materials exhibit strain rate-dependent mechanical behavior to a different extent. The larger strain rate means the loading rate is faster, conceptually the strain rate below 0.001/s is always viewed as the static mechanical manner, which helps us inform the material behavior under more slowly loading cases. For the triaxial compression tests, only the loading rate at 0.001/s corresponding to a static manner was performed in this paper, which is helpfu to understand the volume stress-strain relationship of materials under the pressrue loading. In addition to our research, previous studies on testing the living kidney were also performed in a static uniaxial compression manner on porcine kidney [1], in a tricompression manner with different strain rates on porcine kidney [2] and in a dynamic tricompression manner on human tissues [3]. Further, it should be noted that the existing research about the mechanical behavior of living kidney is limited and there are certain differences in the experimental data obtained by different researchers, thus researchers still need to make experimental efforts to enrich the experimental mechanical database of biomechanics, which forms our motivation of this study.

Mentioned references:

1. Umale S, Deck C, Bourdet N, Dhumane P, Soler L, Marescaux J, et al. Experimental mechanical characterization of abdominal organs: liver, kidney & spleen. Journal of the Mechanical Behavior of Biomedical Materials. 2013; 17:22–33. doi: 10.1016/j.jmbbm.2012.07.010.

2. Farshad M, Barbezat M, Flüeler P, Schmidlin F, Graber P, Niederer P. Material characterization of the pig kidney in relation with the biomechanical analysis of renal trauma. Journal of biomechanics. 1999; 32:417–25. doi: 10.1016/S0021-9290(98)00180-8.

3. Saraf H, Ramesh KT, Lennon AM, Merkle AC, Roberts JC. Mechanical properties of soft human tissues under dynamic loading. J Biomech. 2007;40(9):1960-7.

2. Uniaxial tension tests, why three strain rate values were selected and they compare to physiological values?

Thanks for your comments. As we stated before, various types of materials exhibit strain rate-dependent mechanical behavior but to a different extent. To inform the kidney strain rate-dependent material, three strain rates of 0.001/s, 0.01/s, and 0.1/s were selected in our uniaxial tension tests, the obtained test data can help us know the kidney mechanical behavior under static loading to slowly impact (quasi-static) loading.

The living tissue’s **compression behavior** in different strain rate with range of 0.001/s-3000/s [1-3] and kidney’s **unaxial tension** in a strain rate of 0.001/s corresponding to the static manner [1-2,4] have been performed before. Aa can be seen that the the strain-rate-dependent behavior of kidney **in tension** is limited, which propel us to conduct and complement the experimental data on kidneys. Besides, a damage-dependent visco-elastic constitutive equation was first employed to describe the uniaxial tension behavior in different strain rates, the innovation of this model is that the progressing damage causing the degradation of the kidney during the loading is considered. It should be noted that by using the existing models in **Abaqus**, the strength-reduced behavior in modeling different tests can not be achieved.

Mentioned references:

1. Umale S, Deck C, Bourdet N, Dhumane P, Soler L, Marescaux J, et al. Experimental mechanical characterization of abdominal organs: liver, kidney & spleen. Journal of the Mechanical Behavior of Biomedical Materials. 2013; 17:22–33. doi: 10.1016/j.jmbbm.2012.07.010.

2. Farshad M, Barbezat M, Flüeler P, Schmidlin F, Graber P, Niederer P. Material characterization of the pig kidney in relation with the biomechanical analysis of renal trauma. Journal of biomechanics. 1999; 32:417–25. doi: 10.1016/S0021-9290(98)00180-8.

3. Saraf H, Ramesh KT, Lennon AM, Merkle AC, Roberts JC. Mechanical properties of soft human tissues under dynamic loading. J Biomech. 2007;40(9):1960-7.

4. Karimi A, Shojaei A. Measurement of the Mechanical Properties of the Human Kidney. IRBM. 2017; 38:292–7. doi: 10.1016/j.irbm.2017.08.001.

3. Three strain rates selected here vary by two orders of magnitude and still considered quasistatic by authors.

Thanks for your comments. To inform the kidney strain rate-dependent material, three strain rates of 0.001/s, 0.01/s, and 0.1/s were selected in our uniaxial tension tests. Although the selected strain rates varying by two orders of magnitude belong to the range from static to quasi-static loading, from the experimental results, it appears that the kidney exhibits obvious strain-rate-dependent mechanical behavior in such a strain-rate range.

4. Material and Methods: Contains a paragraph (L137- L149) that seems more like introductory.

Thanks for your comments. To better illustrate the sampling method of porcine kidneys in our test, this paragraph introduces the kidney structural and mechanical characteristics by reviewing previous literature. Thus, this paragraph is necessary and should be located in the section of Material and Methods.

5. Limitations of the study may be clarified.

Thanks for your comments. The limitations of this study were added in the revised paper with the corresponding statement as follows:

Several limitations still exist in this study, which need to be further conducted in future studies. (1) Due to the lack of failure criteria, the current constitutive model cannot predict the elongation at break. (2) The triaxial compression is only performed on the strain rate at 0.001/s corresponding to the static manner, next step, more higher strain rate tests need to be done to present the strain rate-dependent mechanical behavior of the kidney pressure-volume relation.
