# Supplementary material for: Evaluation of the mechanical properties of porcine kidney
Source: PLoS One. 2024 Jul 25;19(7):e0307778. doi: 10.1371/journal.pone.0307778 (PMC11271945; doi:10.1371/journal.pone.0307778)
Supplement: Peer review history

# Peer review: Decision Letter 0

**DOI:** 10.1371/journal.pone.0307778.r001

**Authors:** Nitin Kumar Sharma

23 Feb 2024

PONE-D-24-00404Evaluation of the mechanical properties of porcine kidneyPLOS ONE

Dear Dr. Tan,

Thank you for submitting your manuscript to PLOS ONE. After careful consideration, we feel that it has merit but does not fully meet PLOS ONE’s publication criteria as it currently stands. Therefore, we invite you to submit a revised version of the manuscript that addresses the points raised during the review process.

Please submit your revised manuscript by Apr 08 2024 11:59PM. If you will need more time than this to complete your revisions, please reply to this message or contact the journal office at plosone@plos.org. When you're ready to submit your revision, log on to https://www.editorialmanager.com/pone/ and select the 'Submissions Needing Revision' folder to locate your manuscript file.

Please include the following items when submitting your revised manuscript:

- A rebuttal letter that responds to each point raised by the academic editor and reviewer(s). You should upload this letter as a separate file labeled 'Response to Reviewers'.
- A marked-up copy of your manuscript that highlights changes made to the original version. You should upload this as a separate file labeled 'Revised Manuscript with Track Changes'.
- An unmarked version of your revised paper without tracked changes. You should upload this as a separate file labeled 'Manuscript'.

If you would like to make changes to your financial disclosure, please include your updated statement in your cover letter. Guidelines for resubmitting your figure files are available below the reviewer comments at the end of this letter.

If applicable, we recommend that you deposit your laboratory protocols in protocols.io to enhance the reproducibility of your results. Protocols.io assigns your protocol its own identifier (DOI) so that it can be cited independently in the future. For instructions see: https://journals.plos.org/plosone/s/submission-guidelines#loc-laboratory-protocols. Additionally, PLOS ONE offers an option for publishing peer-reviewed Lab Protocol articles, which describe protocols hosted on protocols.io. Read more information on sharing protocols at https://plos.org/protocols?utm_medium=editorial-email&utm_source=authorletters&utm_campaign=protocols.

We look forward to receiving your revised manuscript.

Kind regards,

Nitin Kumar Sharma, PhD

Academic Editor

PLOS ONE

Journal requirements:

When submitting your revision, we need you to address these additional requirements.

1. Please ensure that your manuscript meets PLOS ONE's style requirements, including those for file naming. The PLOS ONE style templates can be found at

https://journals.plos.org/plosone/s/file?id=wjVg/PLOSOne_formatting_sample_main_body.pdf and

https://journals.plos.org/plosone/s/file?id=ba62/PLOSOne_formatting_sample_title_authors_affiliations.pdf

2. We noticed you have some minor occurrence of overlapping text with the following previous publication(s), which needs to be addressed:

- Material characterization of the pig kidney in relation with the biomechanical analysis of renal trauma - https://doi.org/10.1016/S0021-9290(98)00180-8

- Sample, testing and analysis variables affecting liver mechanical properties: A review - https://doi.org/10.1016/j.actbio.2016.08.055

- A unified viscoelastic model of progressive damage and failure for solid propellants - https://doi.org/10.1016/j.ijplas.2023.103765

- Multiscale viscoelastic constitutive modeling of solid propellants subjected to large deformation - https://doi.org/10.1016/j.ijsolstr.2022.112084

- Constitutive modeling of solid propellants for three dimensional nonlinear finite element analysis - https://doi.org/10.1016/j.ast.2017.06.025

In your revision ensure you cite all your sources (including your own works), and quote or rephrase any duplicated text outside the methods section. Further consideration is dependent on these concerns being addressed.

3. Please note that PLOS ONE has specific guidelines on code sharing for submissions in which author-generated code underpins the findings in the manuscript. In these cases, all author-generated code must be made available without restrictions upon publication of the work. Please review our guidelines at https://journals.plos.org/plosone/s/materials-and-software-sharing#loc-sharing-code and ensure that your code is shared in a way that follows best practice and facilitates reproducibility and reuse.

4. Thank you for stating the following financial disclosure:

“The authors would like to thank China National Nature Science Foundation (Grant no. 11872119).”

Please state what role the funders took in the study.  If the funders had no role, please state: "The funders had no role in study design, data collection and analysis, decision to publish, or preparation of the manuscript."

If this statement is not correct you must amend it as needed.

Please include this amended Role of Funder statement in your cover letter; we will change the online submission form on your behalf.

5. When completing the data availability statement of the submission form, you indicated that you will make your data available on acceptance. We strongly recommend all authors decide on a data sharing plan before acceptance, as the process can be lengthy and hold up publication timelines. Please note that, though access restrictions are acceptable now, your entire data will need to be made freely accessible if your manuscript is accepted for publication. This policy applies to all data except where public deposition would breach compliance with the protocol approved by your research ethics board. If you are unable to adhere to our open data policy, please kindly revise your statement to explain your reasoning and we will seek the editor's input on an exemption. Please be assured that, once you have provided your new statement, the assessment of your exemption will not hold up the peer review process.

6. PLOS requires an ORCID iD for the corresponding author in Editorial Manager on papers submitted after December 6th, 2016. Please ensure that you have an ORCID iD and that it is validated in Editorial Manager. To do this, go to ‘Update my Information’ (in the upper left-hand corner of the main menu), and click on the Fetch/Validate link next to the ORCID field. This will take you to the ORCID site and allow you to create a new iD or authenticate a pre-existing iD in Editorial Manager. Please see the following video for instructions on linking an ORCID iD to your Editorial Manager account: https://www.youtube.com/watch?v=_xcclfuvtxQ.

7. Please include a separate caption for each figure in your manuscript.

Reviewers' comments:

Reviewer's Responses to Questions

**Comments to the Author**

1. Is the manuscript technically sound, and do the data support the conclusions?

The manuscript must describe a technically sound piece of scientific research with data that supports the conclusions. Experiments must have been conducted rigorously, with appropriate controls, replication, and sample sizes. The conclusions must be drawn appropriately based on the data presented. 

Reviewer #1: Partly

Reviewer #2: Yes

**********

2. Has the statistical analysis been performed appropriately and rigorously? 

Reviewer #1: No

Reviewer #2: No

**********

3. Have the authors made all data underlying the findings in their manuscript fully available?

The PLOS Data policy requires authors to make all data underlying the findings described in their manuscript fully available without restriction, with rare exception (please refer to the Data Availability Statement in the manuscript PDF file). The data should be provided as part of the manuscript or its supporting information, or deposited to a public repository. For example, in addition to summary statistics, the data points behind means, medians and variance measures should be available. If there are restrictions on publicly sharing data—e.g. participant privacy or use of data from a third party—those must be specified.

Reviewer #1: Yes

Reviewer #2: Yes

**********

4. Is the manuscript presented in an intelligible fashion and written in standard English?

PLOS ONE does not copyedit accepted manuscripts, so the language in submitted articles must be clear, correct, and unambiguous. Any typographical or grammatical errors should be corrected at revision, so please note any specific errors here.

Reviewer #1: Yes

Reviewer #2: No

**********

5. Review Comments to the Author

Please use the space provided to explain your answers to the questions above. You may also include additional comments for the author, including concerns about dual publication, research ethics, or publication ethics. (Please upload your review as an attachment if it exceeds 20,000 characters)

Reviewer #1: The paper titled "Evaluation of the Mechanical Properties of Porcine Kidney" by Zhao Zhang, Xianglong Tan*, Mengyang Li, Wubuliaisan Ma, Shangjian Zeng, and Yanqing Wu* investigates the mechanical properties of fresh porcine kidneys, focusing on their viscoelastic material behavior. I am having a hard time to figure out the novelty of this paper!

1) The term "fracture" for soft biological tissues does not seem accurate. Maybe rupture or tissue failure would be a better choice.

2) While the introduction well acknowledged the work that has been done so far, I am still confused what has been missing in the field that you try to address in this paper? just different strain rates? just different material models? I do not think all this is original enough for a publication!

3) What made you to choose such different strain rates? from 1 mm/min to 100 mm/s? you sure that is not a typo?

4) Abaqus has all these material models and can calculate these properties so why did you add to this?

5) Why even we need to know about the "fracture" of this tissue? You do not have any information in the paper that why do we need to know about this?

6) Could not find any limitation section?

Reviewer #2: Zhang et al show an interesting study on physical characteristics of porcine kidney based on experimental data (on tension, compression and relaxation) supported by constitutive modeling. However, poor presented/substantiated methodology and findings make it difficult to understand and impair overall impact of the work. Below are few comments to guide authors.

1. Abstract too long and conclusions may be made concise

2. Triaxial compression tests, how such a loading rate was selected and it compares to physiological values??

3. Uniaxial tension tests, why three strain rate values were selected and they compare to physiological values?

4. Relaxation tests: Why 20 mins and 3 mm (not less or more) for relaxation tests?

5. A lot of raw data is shown in but the relation among results obtained by different techniques (including effect of strain rate), and statistical caparison is missing (maybe bar chart?)

6. L197: Where did you calculate elastic modulus and which elastic modulus (E or K)?

7. L204: The justification for constitutive model selection is not provided, and physical significance of the selected parameters may be provided for better interpretation of the findings.

8. L266, L271,L272: Which strength?

9. Figs title may elaborate the fig to guide the reader.

10. Fig.1: Color contrast may be adjusted to improve contrast; Fig.2 & 3: units missing

11. Discussion fails to compare obtained modulus values to existing values in literature (e.g. ~ 1-10kPa range for Kidney). While authors obtain ~30kPa, such difference exists may be discussed…

12. Introduction may mention about the difference among tissues from different species….

**********

6. PLOS authors have the option to publish the peer review history of their article (what does this mean?). If published, this will include your full peer review and any attached files.

If you choose “no”, your identity will remain anonymous but your review may still be made public.

**Do you want your identity to be public for this peer review?** For information about this choice, including consent withdrawal, please see our Privacy Policy.

Reviewer #1: No

Reviewer #2: No

**********

[NOTE: If reviewer comments were submitted as an attachment file, they will be attached to this email and accessible via the submission site. Please log into your account, locate the manuscript record, and check for the action link "View Attachments". If this link does not appear, there are no attachment files.]

While revising your submission, please upload your figure files to the Preflight Analysis and Conversion Engine (PACE) digital diagnostic tool, https://pacev2.apexcovantage.com/. PACE helps ensure that figures meet PLOS requirements. To use PACE, you must first register as a user. Registration is free. Then, login and navigate to the UPLOAD tab, where you will find detailed instructions on how to use the tool. If you encounter any issues or have any questions when using PACE, please email PLOS at figures@plos.org. Please note that Supporting Information files do not need this step.

# Author comment: Author response to Decision Letter 0

**DOI:** 10.1371/journal.pone.0307778.r002

18 Apr 2024

Thank you very much. We have replied all the modification suggestions in a separate file and named it as “Response to Reviewers”

Attachment Submitted filename: Response to Reviewers.docx

# Peer review: Decision Letter 1

**DOI:** 10.1371/journal.pone.0307778.r003

**Authors:** Nitin Kumar Sharma

29 May 2024

PONE-D-24-00404R1Evaluation of the mechanical properties of porcine kidneyPLOS ONE

Dear Dr. Tan,

Thank you for submitting your manuscript to PLOS ONE. After careful consideration, we feel that it has merit but does not fully meet PLOS ONE’s publication criteria as it currently stands. Therefore, we invite you to submit a revised version of the manuscript that addresses the points raised during the review process.

Please submit your revised manuscript by Jul 13 2024 11:59PM. If you will need more time than this to complete your revisions, please reply to this message or contact the journal office at plosone@plos.org. When you're ready to submit your revision, log on to https://www.editorialmanager.com/pone/ and select the 'Submissions Needing Revision' folder to locate your manuscript file.

Please include the following items when submitting your revised manuscript:

- A rebuttal letter that responds to each point raised by the academic editor and reviewer(s). You should upload this letter as a separate file labeled 'Response to Reviewers'.
- A marked-up copy of your manuscript that highlights changes made to the original version. You should upload this as a separate file labeled 'Revised Manuscript with Track Changes'.
- An unmarked version of your revised paper without tracked changes. You should upload this as a separate file labeled 'Manuscript'.

If you would like to make changes to your financial disclosure, please include your updated statement in your cover letter. Guidelines for resubmitting your figure files are available below the reviewer comments at the end of this letter.

If applicable, we recommend that you deposit your laboratory protocols in protocols.io to enhance the reproducibility of your results. Protocols.io assigns your protocol its own identifier (DOI) so that it can be cited independently in the future. For instructions see: https://journals.plos.org/plosone/s/submission-guidelines#loc-laboratory-protocols. Additionally, PLOS ONE offers an option for publishing peer-reviewed Lab Protocol articles, which describe protocols hosted on protocols.io. Read more information on sharing protocols at https://plos.org/protocols?utm_medium=editorial-email&utm_source=authorletters&utm_campaign=protocols.

We look forward to receiving your revised manuscript.

Kind regards,

Nitin Kumar Sharma, PhD

Academic Editor

PLOS ONE

Journal Requirements:

Please review your reference list to ensure that it is complete and correct. If you have cited papers that have been retracted, please include the rationale for doing so in the manuscript text, or remove these references and replace them with relevant current references. Any changes to the reference list should be mentioned in the rebuttal letter that accompanies your revised manuscript. If you need to cite a retracted article, indicate the article’s retracted status in the References list and also include a citation and full reference for the retraction notice.

**Additional Editor Comments:**

Dear Authors,

Thanks for your efforts in revising the manuscript. After careful observation and looking in to the reviewers comments, I found that your findings are valuable in the field of biomedical science. However, there are few comments raised by the reviewers. You are welcome to address these comments and submit the revised version for further consideration. The comments are as given below;

Since the revised version don’t seem to provide sufficient literature references indicating physiological values that has been used by authors as a basis for their experiments and the edits made by authors based on suggestions seem unclear in the revised manuscript version (e.g edits in response to each comment need to be clearly indicated by corresponding Line numbers in manuscript), I would seek a major revision. The below points are merely indicative, and the authors should carefully revise the manuscript to significantly improve the reader’s confidence.

1. Triaxial compression tests, how such a loading rate was selected and it compares to physiological values??

“The strain rate equal or below 0.001/s can be viewed as static manner, …”

Is it percent strain rate may be clarified?

2. Uniaxial tension tests, why three strain rate values were selected and they compare to physiological values?

3. Three three strain rates selected here vary by two orders of magnitude and still considered quasistatic by authors.

4. Material and Methods: Contains a paragraph (L137- L149) that seems more like introductory.

5. Limitations of the study may be clarified

[Note: HTML markup is below. Please do not edit.]

Reviewers' comments:

Reviewer's Responses to Questions

**Comments to the Author**

1. If the authors have adequately addressed your comments raised in a previous round of review and you feel that this manuscript is now acceptable for publication, you may indicate that here to bypass the “Comments to the Author” section, enter your conflict of interest statement in the “Confidential to Editor” section, and submit your "Accept" recommendation.

Reviewer #1: All comments have been addressed

Reviewer #2: (No Response)

**********

2. Is the manuscript technically sound, and do the data support the conclusions?

The manuscript must describe a technically sound piece of scientific research with data that supports the conclusions. Experiments must have been conducted rigorously, with appropriate controls, replication, and sample sizes. The conclusions must be drawn appropriately based on the data presented. 

Reviewer #1: Yes

Reviewer #2: No

**********

3. Has the statistical analysis been performed appropriately and rigorously? 

Reviewer #1: (No Response)

Reviewer #2: No

**********

4. Have the authors made all data underlying the findings in their manuscript fully available?

The PLOS Data policy requires authors to make all data underlying the findings described in their manuscript fully available without restriction, with rare exception (please refer to the Data Availability Statement in the manuscript PDF file). The data should be provided as part of the manuscript or its supporting information, or deposited to a public repository. For example, in addition to summary statistics, the data points behind means, medians and variance measures should be available. If there are restrictions on publicly sharing data—e.g. participant privacy or use of data from a third party—those must be specified.

Reviewer #1: (No Response)

Reviewer #2: Yes

**********

5. Is the manuscript presented in an intelligible fashion and written in standard English?

PLOS ONE does not copyedit accepted manuscripts, so the language in submitted articles must be clear, correct, and unambiguous. Any typographical or grammatical errors should be corrected at revision, so please note any specific errors here.

Reviewer #1: (No Response)

Reviewer #2: Yes

**********

6. Review Comments to the Author

Please use the space provided to explain your answers to the questions above. You may also include additional comments for the author, including concerns about dual publication, research ethics, or publication ethics. (Please upload your review as an attachment if it exceeds 20,000 characters)

Reviewer #1: (No Response)

Reviewer #2: Since the revised version don’t seem to provide sufficient literature references indicating physiological values that has been used by authors as a basis for their experiments and the edits made by authors based on suggestions seem unclear in the revised manuscript version (e.g edits in response to each comment need to be clearly indicated by corresponding Line numbers in manuscript), I would seek a major revision. The below points are merely indicative, and hence, the authors should carefully revise the manuscript to significantly improve the reader’s confidence.

1. Triaxial compression tests, how such a loading rate was selected and

it compares to physiological values??

“The strain rate equal or below 0.001/s can be viewed as static manner, …”

Is it percent strain rate may be clarified?

2. "Uniaxial tension tests, why three strain rate values were selected

and they compare to physiological values?"

Three three strain rates selected here vary by two orders of magnitude but still considered quasistatic by authors.

3. Material and Methods: Contains a paragraph (L137- L149) that seems more like introductory.

4. Limitations of the study may be clarified

**********

7. PLOS authors have the option to publish the peer review history of their article (what does this mean?). If published, this will include your full peer review and any attached files.

If you choose “no”, your identity will remain anonymous but your review may still be made public.

**Do you want your identity to be public for this peer review?** For information about this choice, including consent withdrawal, please see our Privacy Policy.

Reviewer #1: No

Reviewer #2: No

**********

[NOTE: If reviewer comments were submitted as an attachment file, they will be attached to this email and accessible via the submission site. Please log into your account, locate the manuscript record, and check for the action link "View Attachments". If this link does not appear, there are no attachment files.]

While revising your submission, please upload your figure files to the Preflight Analysis and Conversion Engine (PACE) digital diagnostic tool, https://pacev2.apexcovantage.com/. PACE helps ensure that figures meet PLOS requirements. To use PACE, you must first register as a user. Registration is free. Then, login and navigate to the UPLOAD tab, where you will find detailed instructions on how to use the tool. If you encounter any issues or have any questions when using PACE, please email PLOS at figures@plos.org. Please note that Supporting Information files do not need this step.

Attachment Submitted filename: Review report_PONE-D-00404_R1.docx

# Author comment: Author response to Decision Letter 1

**DOI:** 10.1371/journal.pone.0307778.r004

25 Jun 2024

Response to Reviewer

General comments:

Since the revised version don’t seem to provide sufficient literature references indicating physiological values that has been used by authors as a basis for their experiments and the edits made by authors based on suggestions seem unclear in the revised manuscript version (e.g edits in response to each comment need to be clearly indicated by corresponding Line numbers in manuscript), I would seek a major revision. The below points are merely indicative, and the authors should carefully revise the manuscript to significantly improve the reader’s confidence. 

Reply:

Thanks to the Reviewer for their comments concerning our manuscript entitled “Evaluation of the mechanical properties of porcine kidney” (Manuscript Number: PONE-D-24-00404R1). Those comments are all valuable and helpful for further improving our paper and as the important guiding significance to our researchers. We have studied comments carefully and have made correction which we hope meet with approval. Revised portion are marked in bold and blue fonts in the revised manuscript. The responses to the comments by the reviewer are as follows.

1. Triaxial compression tests, how such a loading rate was selected and it compares to physiological values? “The strain rate equal or below 0.001/s can be viewed as static manner, …” , Is it percent strain rate may be clarified?

Thanks for your comments. From the mechanical concept, the strain rate is the change in strain (deformation) of a material with respect to time, many materials exhibit strain rate-dependent mechanical behavior to a different extent. The larger strain rate means the loading rate is faster, conceptually the strain rate below 0.001/s is always viewed as the static mechanical manner, which helps us inform the material behavior under more slowly loading cases. For the triaxial compression tests, only the loading rate at 0.001/s corresponding to a static manner was performed in this paper, which is helpfu to understand the volume stress-strain relationship of materials under the pressrue loading. In addition to our research, previous studies on testing the living kidney were also performed in a static uniaxial compression manner on porcine kidney [1], in a tricompression manner with different strain rates on porcine kidney [2] and in a dynamic tricompression manner on human tissues [3]. Further, it should be noted that the existing research about the mechanical behavior of living kidney is limited and there are certain differences in the experimental data obtained by different researchers, thus researchers still need to make experimental efforts to enrich the experimental mechanical database of biomechanics, which forms our motivation of this study. 

Mentioned references:

1. Umale S, Deck C, Bourdet N, Dhumane P, Soler L, Marescaux J, et al. Experimental mechanical characterization of abdominal organs: liver, kidney & spleen. Journal of the Mechanical Behavior of Biomedical Materials. 2013; 17:22–33. doi: 10.1016/j.jmbbm.2012.07.010.

2. Farshad M, Barbezat M, Flüeler P, Schmidlin F, Graber P, Niederer P. Material characterization of the pig kidney in relation with the biomechanical analysis of renal trauma. Journal of biomechanics. 1999; 32:417–25. doi: 10.1016/S0021-9290(98)00180-8.

3. Saraf H, Ramesh KT, Lennon AM, Merkle AC, Roberts JC. Mechanical properties of soft human tissues under dynamic loading. J Biomech. 2007;40(9):1960-7.

2. Uniaxial tension tests, why three strain rate values were selected and they compare to physiological values?

Thanks for your comments. As we stated before, various types of materials exhibit strain rate-dependent mechanical behavior but to a different extent. To inform the kidney strain rate-dependent material, three strain rates of 0.001/s, 0.01/s, and 0.1/s were selected in our uniaxial tension tests, the obtained test data can help us know the kidney mechanical behavior under static loading to slowly impact (quasi-static) loading. 

The living tissue’s compression behavior in different strain rate with range of 0.001/s-3000/s [1-3] and kidney’s unaxial tension in a strain rate of 0.001/s corresponding to the static manner [1-2,4] have been performed before. Aa can be seen that the the strain-rate-dependent behavior of kidney in tension is limited, which propel us to conduct and complement the experimental data on kidneys. Besides, a damage-dependent visco-elastic constitutive equation was first employed to describe the uniaxial tension behavior in different strain rates, the innovation of this model is that the progressing damage causing the degradation of the kidney during the loading is considered. It should be noted that by using the existing models in Abaqus, the strength-reduced behavior in modeling different tests can not be achieved. 

Mentioned references:

1. Umale S, Deck C, Bourdet N, Dhumane P, Soler L, Marescaux J, et al. Experimental mechanical characterization of abdominal organs: liver, kidney & spleen. Journal of the Mechanical Behavior of Biomedical Materials. 2013; 17:22–33. doi: 10.1016/j.jmbbm.2012.07.010.

2. Farshad M, Barbezat M, Flüeler P, Schmidlin F, Graber P, Niederer P. Material characterization of the pig kidney in relation with the biomechanical analysis of renal trauma. Journal of biomechanics. 1999; 32:417–25. doi: 10.1016/S0021-9290(98)00180-8.

3. Saraf H, Ramesh KT, Lennon AM, Merkle AC, Roberts JC. Mechanical properties of soft human tissues under dynamic loading. J Biomech. 2007;40(9):1960-7.

4. Karimi A, Shojaei A. Measurement of the Mechanical Properties of the Human Kidney. IRBM. 2017; 38:292–7. doi: 10.1016/j.irbm.2017.08.001.

3. Three strain rates selected here vary by two orders of magnitude and still considered quasistatic by authors. 

Thanks for your comments. To inform the kidney strain rate-dependent material, three strain rates of 0.001/s, 0.01/s, and 0.1/s were selected in our uniaxial tension tests. Although the selected strain rates varying by two orders of magnitude belong to the range from static to quasi-static loading, from the experimental results, it appears that the kidney exhibits obvious strain-rate-dependent mechanical behavior in such a strain-rate range. 

4. Material and Methods: Contains a paragraph (L137- L149) that seems more like introductory.

Thanks for your comments. To better illustrate the sampling method of porcine kidneys in our test, this paragraph introduces the kidney structural and mechanical characteristics by reviewing previous literature. Thus, this paragraph is necessary and should be located in the section of Material and Methods.

5. Limitations of the study may be clarified.

Thanks for your comments. The limitations of this study were added in the revised paper with the corresponding statement as follows:

Several limitations still exist in this study, which need to be further conducted in future studies. (1) Due to the lack of failure criteria, the current constitutive model cannot predict the elongation at break. (2) The triaxial compression is only performed on the strain rate at 0.001/s corresponding to the static manner, next step, more higher strain rate tests need to be done to present the strain rate-dependent mechanical behavior of the kidney pressure-volume relation.

Attachment Submitted filename: Response to Reviewers.docx

# Editor report: Decision Letter 2

**DOI:** 10.1371/journal.pone.0307778.r005

**Authors:** Nitin Kumar Sharma

11 Jul 2024

Evaluation of the mechanical properties of porcine kidney

PONE-D-24-00404R2

Dear Dr. Tan,

We’re pleased to inform you that your manuscript has been judged scientifically suitable for publication and will be formally accepted for publication once it meets all outstanding technical requirements.

Within one week, you’ll receive an e-mail detailing the required amendments. When these have been addressed, you’ll receive a formal acceptance letter and your manuscript will be scheduled for publication.

An invoice will be generated when your article is formally accepted. Please note, if your institution has a publishing partnership with PLOS and your article meets the relevant criteria, all or part of your publication costs will be covered. Please make sure your user information is up-to-date by logging into Editorial Manager at Editorial Manager® and clicking the ‘Update My Information' link at the top of the page. If you have any questions relating to publication charges, please contact our Author Billing department directly at authorbilling@plos.org.

If your institution or institutions have a press office, please notify them about your upcoming paper to help maximize its impact. If they’ll be preparing press materials, please inform our press team as soon as possible -- no later than 48 hours after receiving the formal acceptance. Your manuscript will remain under strict press embargo until 2 pm Eastern Time on the date of publication. For more information, please contact onepress@plos.org.

Kind regards,

Nitin Kumar Sharma, PhD

Academic Editor

PLOS ONE

Additional Editor Comments (optional):

Reviewers' comments:

# Editor report: Acceptance letter

**DOI:** 10.1371/journal.pone.0307778.r006

**Authors:** Nitin Kumar Sharma

15 Jul 2024

PONE-D-24-00404R2 

PLOS ONE

Dear Dr. Tan, 

I'm pleased to inform you that your manuscript has been deemed suitable for publication in PLOS ONE. Congratulations! Your manuscript is now being handed over to our production team.

At this stage, our production department will prepare your paper for publication. This includes ensuring the following:

* All references, tables, and figures are properly cited

* All relevant supporting information is included in the manuscript submission,

* There are no issues that prevent the paper from being properly typeset

If revisions are needed, the production department will contact you directly to resolve them. If no revisions are needed, you will receive an email when the publication date has been set. At this time, we do not offer pre-publication proofs to authors during production of the accepted work. Please keep in mind that we are working through a large volume of accepted articles, so please give us a few weeks to review your paper and let you know the next and final steps. 

Lastly, if your institution or institutions have a press office, please let them know about your upcoming paper now to help maximize its impact. If they'll be preparing press materials, please inform our press team within the next 48 hours. Your manuscript will remain under strict press embargo until 2 pm Eastern Time on the date of publication. For more information, please contact onepress@plos.org.

If we can help with anything else, please email us at customercare@plos.org.

Thank you for submitting your work to PLOS ONE and supporting open access. 

Kind regards, 

PLOS ONE Editorial Office Staff

on behalf of

Dr. Nitin Kumar Sharma 

Academic Editor

PLOS ONE
